# Neurocognitive Dynamics of Prosodic Salience over Semantics during Explicit and Implicit Processing of Basic Emotions in Spoken Words

**DOI:** 10.3390/brainsci12121706

**Published:** 2022-12-12

**Authors:** Yi Lin, Xinran Fan, Yueqi Chen, Hao Zhang, Fei Chen, Hui Zhang, Hongwei Ding, Yang Zhang

**Affiliations:** 1 Speech-Language-Hearing Center, School of Foreign Languages, Shanghai Jiao Tong University, Shanghai 200240, China; 2 School of Foreign Languages and Literature, Shandong University, Jinan 250100, China; 3 School of Foreign Languages, Hunan University, Changsha 410012, China; 4 School of International Education, Shandong University, Jinan 250100, China; 5 Department of Speech-Language-Hearing Science & Masonic Institute for the Developing Brain, University of Minnesota, Minneapolis, MN 55455, USA

**Keywords:** emotional speech processing, N400, late positive response, ITPC, ERSP

## Abstract

How language mediates emotional perception and experience is poorly understood. The present event-related potential (ERP) study examined the explicit and implicit processing of emotional speech to differentiate the relative influences of communication channel, emotion category and task type in the prosodic salience effect. Thirty participants (15 women) were presented with spoken words denoting happiness, sadness and neutrality in either the prosodic or semantic channel. They were asked to judge the emotional content (explicit task) and speakers’ gender (implicit task) of the stimuli. Results indicated that emotional prosody (relative to semantics) triggered larger N100, P200 and N400 amplitudes with greater delta, theta and alpha inter-trial phase coherence (ITPC) and event-related spectral perturbation (ERSP) values in the corresponding early time windows, and continued to produce larger LPC amplitudes and faster responses during late stages of higher-order cognitive processing. The relative salience of prosodic and semantics was modulated by emotion and task, though such modulatory effects varied across different processing stages. The prosodic salience effect was reduced for sadness processing and in the implicit task during early auditory processing and decision-making but reduced for happiness processing in the explicit task during conscious emotion processing. Additionally, across-trial synchronization of delta, theta and alpha bands predicted the ERP components with higher ITPC and ERSP values significantly associated with stronger N100, P200, N400 and LPC enhancement. These findings reveal the neurocognitive dynamics of emotional speech processing with prosodic salience tied to stage-dependent emotion- and task-specific effects, which can reveal insights into understanding language and emotion processing from cross-linguistic/cultural and clinical perspectives.

## 1. Introduction

### 1.1. Sensory Dominance Effects: Theoretical Importance and Methodological Concerns

Emotion plays an essential role in successful interpersonal communication. Humans show how they feel through what they say (i.e., linguistic content) and how they say it (i.e., paralinguistic information). One important theoretical contention centering around multisensory emotional speech processing is whether a certain sensory channel is more perceptually dominant over others, which is referred to as the channel (sensory) dominance effect [1,2]. A focus on channel dominance, especially the role of prosody, in emotional speech processing is crucial for understanding the developmental trajectory and functional impairments of speech, language and hearing abilities. Studies have shown that infants are highly sensitive to the prosodic aspects of early language input that provides socio-affective foundation for language acquisition [3,4]. For individuals with typical language skills, prosody is a salient part of multisensory speech communication [1,2]. In aging, emotional prosody is also difficult for individuals with hearing loss and cognitive decline [5,6,7]. Various clinical populations struggle with emotional speech processing, including patients with schizophrenia and autism [8,9].

While some studies observed the predominance of auditory prosodic cues over verbal content in emotional speech perception [10,11], there is also evidence pointing to a perceptual bias towards semantic content [2,12]. These empirical discrepancies in behavioral investigations may be related to differences in language and cultural background across studies. Given the cross-linguistic differences and socio-cultural nature of decoding and encoding emotions, what is considered a normal pitch or rhythm in a tonal language (e.g., Mandarin Chinese) may be considered excessive in a non-tonal language (e.g., Italian) and vice versa [13]. Notably, those studies supporting a semantic dominance effect are largely based on data collected in western countries (e.g., Germany and Canada) with a non-tonal language background and a low-context culture [14], in which interlocutors tend to rely heavily on verbal messages during speech communication. It remains to be tested to what extent the existing findings can be generalized to a different socio-contextual background, such as a high-context culture, where nonverbal information and interpersonal relationships are more important [15]. For studies investigating the neural underpinnings of emotional semantics and prosody processing, extensive efforts have been made to specify the related brain structures using functional neuroimaging [16,17,18,19,20]. Relatively fewer studies have explored the underlying time course using neurophysiological techniques with fine temporal resolution (e.g., electroencephalogram) [21].

Though conventional ERP waveform analysis can shed light on the event-locked regularities of brain dynamics based on time-domain information averaged across trials, it may underestimate trial-by-trial response variability in the time-frequency domain [22,23,24]. A line of studies have applied time-frequency analyses to explore the time-locked and phase-locked neural substrates of auditory processing [23,25,26,27,28,29], though these investigations were often conducted with non-emotional stimuli. In these studies, event-related cortical oscillations can be evaluated through inter-trial phase coherence (ITPC) and event-related spectral perturbation (ERSP) in five frequency bands, including delta (1–4 Hz), theta (4–8 Hz), alpha (8–12 Hz), beta (12–30 Hz) and gamma (over 30 Hz). Higher ITPC values suggest better phase alignment of cortical oscillations, while smaller values indicate poorer consistency or larger neural “jittering” across trials [30]. Higher ERSP values indicate greater changes in EEG spectral power (in dB) as a function of frequency over the time course of the ERP. Results suggested that EEG oscillations, especially delta, theta and alpha ITPC or ERSP, forms a crucial basis for the neural generation of auditory ERP [23,28,31,32]. By contrast, time-frequency analyses of vocal emotion processing are sparse with even less attention on the relationship between ERP waveforms and neural oscillations [33,34]. The combination of ERP waveform and time-frequency analyses in the current study may provide meaningful insights into the underlying neural mechanisms of emotional speech processing.

In light of the theoretical and methodological issues, the primary focus of this work is to examine the temporal dynamics of emotional speech processing using the event-related potential (ERP) measure with waveform and time-frequency analyses. Importantly, we strived to characterize the neurobehavioral representations of channel dominance effects with consideration of emotional category and task type, which can contribute to the understanding of existing discrepancies in previous literature. Since we based our study on a Mandarin Chinese context, the tonal language background enabled us to investigate how pitch variations denoting lexical meaning alone are processed differently from those communicating emotional and linguistic meaning simultaneously at early and late stages. The high-context East Asian setting also allowed for a new cultural perspective on the neurobehavioral distinctions of verbal and nonverbal processing.

### 1.2. Effects of Communication Channel on Multi-Stage Processing of Emotional Speech

Decoding emotional information in speech occurs rapidly, involving a multilayered process that contains temporally and functionally distinct processing stages [16,35,36]. According to Schirmer and Kotz [37], there are three stages for emotional speech processing: (1) analyzing the acoustic features in vocalizations, (2) deriving the emotional salience from a set of acoustic signals, and (3) integrating emotional significance to higher-order cognitive processes. The first two stages have largely been studied with the N100 and P200 components using the ERP technique, and the third stage can be probed with the N400, late positive component (LPC) as well as behavioral measures [21,38,39,40,41,42]. However, it remains unclear how the relative salience of semantic versus prosodic channels unfolds across the different emotional speech processing stages.

There has been divided attention in the literature on prosodic and semantic aspects of emotional speech processing. For instance, the 3-stage model by Schirmer and Kotz [37] characterizes the prosodic aspect of processing for emotional speech, and many studies supporting the model focused on emotional prosody by employing non-linguistic affective vocalizations or pseudo-words/sentences [36,38,43,44,45]. Some studies applied a cross-splicing paradigm to temporally control when prosodic cues became available to the listener by artificially introducing discrepancies between verbal and nonverbal messages [36,40,46]. Likewise, ERP studies on semantic processing of emotional words often chose the visual modality for stimulus presentation without considering emotional prosody in speech [47,48,49,50,51].

Some limitations in the existing research may have prevented us from gaining a comprehensive understanding of the relationship between the two speech channels. One previous ERP investigation substantiated the predominance of semantics over prosody during deviance detection in emotional contexts [36]. However, since the effect was observed based on sentence-level stimuli, its generalizability to other linguistic representations (e.g., word) warrants further examination. It also remains to be tested whether the effect occurs based on semantic mismatch alone or depends on integrative semantic and prosodic processing. In addition, the speech stimuli especially those with unintelligible semantic content are somewhat disassociated from what we are usually faced with in daily communication. Recent behavioral studies attempted to address the joint multi-sensory multi-channel processing of emotional speech, but the behavioral data (including accuracy and reaction time) could not easily separate the final decision-making stage from the earlier processing stages [2,52,53].

### 1.3. Effects of Emotion Category on Emotional Speech Processing

In addition to the relative salience of the communication channels, emotional speech processing is subject to a number of influential factors. One key issue is whether emotional and non-emotional signals can be distinguished from each other automatically at an early stage and if so, exactly when they start to be differentiated. There is cumulative evidence that emotional stimuli elicited larger auditory ERP responses and greater neural synchronization (esp. in the delta and theta band) than neutral stimuli [36,37,54,55]. This can be explained by the evolutionary significance of affective signals, which leads to increased automatic attentional capture and prioritized processing strategies relative to neutral stimuli [36,56]. However, findings are mixed concerning how early the significant differentiation occurs. The processing of emotional speech is generally thought to diverge from that of neutral speech around 200 milliseconds (ms) post stimulus presentation [21,37,57,58], but there is also evidence indicating the distinction as early as 100 ms [43].

A second issue is how different categories of emotion in speech are distinguished from one another. According to the differential emotion theory, a set of emotions (e.g., joy, interest, sadness, anger, fear, disgust) are distinguishable in neurochemical processes, expressive behaviors and subjective experiences [59]. These discrete emotions can also be described in a two-dimensional space with regard to their valence and arousal. Empirical evidence has shown how the two dimensions can influence emotion perception at different processing stages. For example, Paulmann, Bleichner and Kotz [45] found that valence-relevant information can be reliably deciphered at both early and late processing stages, while arousal is more robustly decoded during the late processing stage. Although there tends to be perceptual bias towards positive and high-arousing stimuli, these valence- and arousal-dependent processing patterns have not been conclusively established [60,61]. Some studies have shown valence- and arousal- independent emotion processing [36,62]. Notably, neurophysiological studies on emotional speech processing have generally taken valence attributes into account in stimulus design while disregarding the possible role of arousal. One example is that happiness and anger are often chosen as the two contrasting emotions [38,39], but both of them are high arousing emotions despite a distinction in valence. Thus, the relative influences of valence and arousal on emotional speech processing need to be further investigated with the inclusion of more emotional categories.

### 1.4. Effects of Task Type on Emotional Speech Processing

A third factor is the experimental task. Task focuses can be changed under different types of tasks. In explicit emotion processing tasks, participants are required to evaluate the emotional content (e.g., valence and arousal attributes) of the stimuli. By contrast, attention in implicit tasks is diverted from the emotional attributes of the stimuli and focused on other informational dimensions [63]. Differentiated effects of attention have been found on several ERP components, with increased attention evoking enhanced N100 and N400 but diminished P200 amplitudes [37,64,65,66,67]. Early and late processing of emotional speech can also be modulated by task difficulty/cognitive efforts. Increased task complexity leads to enhanced early auditory ERP responses (e.g., more negative N100, more positive P200) and neural synchrony [39,68,69,70] but reduced brain responses and poorer behavioral performances in the post-perceptual processing stage [38,71,72]. Though some studies indicated that task types can modulate modality- (e.g., visual vs. auditory) or category-specific emotion processing [2,73,74,75], this is not always the case probably due to varying task requirements [45]. To what extent the observed effects of channel and emotion in speech processing can be generalized across different task types warrants further examination.

### 1.5. The Present Study

The present study aimed to examine the neurobehavioral effects of communication channel, emotional category and task type as emotional speech processing unfolded in time. Two basic emotions (i.e., happiness and sadness) and neutrality [76] were tested, and these emotional categories can be distinguished from one another on both valence and arousal scales. Emotional information was conveyed through either the prosodic or semantic channel, which constituted two types of experimental stimuli, namely semantically neutral words spoken in emotional intonations and emotional words spoken in neutral prosody. Participants were asked to identify these emotional stimuli in explicit (i.e., emotion identification tasks) and implicit (i.e., gender identification tasks) conditions. We measured N100, P200, N400, LPC and their associated cortical oscillatory activities to characterize sensory processing of acoustic signals, initial decoding of emotional significance, and early stages of cognitive evaluation. Delta, theta and alpha ITPC and ERSP were selected for evaluation as these frequency band oscillations could reflect salience detection, emotional significance and attentional modulation [55], and could better predict auditory ERP responses [23,28,31]. We also recorded accuracy and reaction time data from stimulus offset to show emotional speech processing in the decision-making stage.

Based on previous studies revealing the effects of channel, emotion and task on emotional speech processing and the relationships among different neurological and behavioral measures, we developed the following hypotheses:

- First, we expected to find ERP and behavioral differentiation of emotional prosody and semantics given the channel (prosodic) dominance effects observed in our recent studies based on a tonal language and high-context culture [2,53].
- Second, we predicted that emotional stimuli would be distinguished from the neutral ones [36,56], and differences would also be found between specific emotion types (i.e., happy and sad) [45].
- Third, task types would modulate brain and behavioral responses during emotional speech processing, since our task instructions would lead to differences in task focuses and difficulty [71,74].
- Finally, we hypothesized that neural oscillation data could be potential indicators of auditory ERP responses [23,28,31]. However, processing patterns were likely to vary across the neurophysiological and behavioral indices since the adopted measures were not conceptually equivalent [22,23].

Findings from the present study will contribute new data to the multi-stage model of emotional speech processing and reveal insights to research on emotion cognition from cross-linguistic/cultural and clinical perspectives.

## 2. Materials and Methods

### 2.1. Participants

Thirty volunteers (15 females and 15 males) were recruited to take part in this experiment through an online campus advertisement. Participants averaged 23.1 (SD = 2.2) years in age and had received an average of 16.6 (SD = 2.2) years of formal school education. All participants were native speakers of Mandarin Chinese with no medical history of speech, language and hearing disorders or neurological problems. All had normal or corrected-to-normal vision and normal hearing in standard audiometric assessment (≤20 dB HL for 0.25-, 0.5-, 1-, 2-, 4-, and 8-kHz pure tones) [77]. All were studying at SJTU as undergraduate or graduate students at the time of testing and were non-musicians without formal musical training in the past five years and less than two years of musical training prior to that [78]. Written informed consent was obtained from all participants, who were paid for their time and involvement.

### 2.2. Stimuli

The stimuli contained two sets of disyllabic words in Mandarin Chinese spoken by a female and a male professional speaker. Each auditory stimulus conveyed one of the two basic emotions (i.e., happiness and sadness) [76] or neutrality in either the prosodic or semantic channel. There were altogether 180 spoken words in each stimulus set/communication channel, in which the number of words was balanced between the two speakers (i.e.,90 words for each speaker), and among the three emotional categories (i.e.,60 words for each emotion). Specifically, for the prosodic set, 60 semantically neutral concrete nouns were spoken in happy, neutral and sad prosody, respectively. For the semantic set, words were spoken in a neutral tone of voice and conveyed emotional information in verbal content, including 60 adjectives with happy semantics, 60 with sad semantics, and 60 with neutral semantics. Most words and their frequencies were taken from A Dictionary of the Frequency of Commonly Used Modern Chinese Words (Alphabetical sequence section) [79]. The semantic word set had higher word frequency than the prosodic set (*t*(394) = −3.67, *p* < 0.001). See Supplemental Tables S1 and S2 for the list of included words for prosodic and semantic stimuli, respectively. All auditory stimuli were normalized in intensity (at 70 dB) using Praat (version 6.1.41) [80]. The duration and mean f0 measures of the prosodic and semantic stimuli are summarized in Tables S3 and S4 in Supplemental Materials, respectively. The spectral images of the auditory stimuli are illustrated in Figure S1 in Supplemental Materials (Part II).

The stimuli were uttered by two native speakers (one woman and one man) of Mandarin Chinese in a quiet laboratory setting, and digitized onto a Macbook Pro computer with AVID Mbox Mini at a sampling rate of 44,100 kHz with a 16-bit resolution. Each word was portrayed three times by the two speakers, and the best ones were selected according to the results of a norming study. In the norming test, forty adult native speakers of Mandarin Chinese (20 women and 20 men, Mean _age_ = 23.0, SD = 3.4) who did not participate in the current research were invited to perceptually validate the experimental stimuli using Praat [80]. These raters were randomly assigned to one of the two gender-balanced groups (20 raters, 10 women in each group). One group of subjects were asked to rate the word familiarity on a 7-point Likert scale (1 = not familiar, 7 = very familiar) and identify the emotional category of each prosodic and semantic stimulus. The other group of subjects were asked to rate the emotional arousal of each stimulus on a 7-point Likert scale (1 = low, 7 = high). Only words with an average rating of >5 for familiarity and over 85% identification accuracy for emotional categories were included in the present experiment. The mean familiarity rating, identification accuracy and emotional arousal of the finally included word stimuli are shown in Tables S5 and S6 in Supplemental Materials. The familiarity rating did not differ between the prosodic and semantic word sets and no significant difference was found in accuracy and arousal for words in the same emotion category between the two channels (all *p* > 0.05).

### 2.3. Procedure

During the electroencephalograph (EEG) recording session, participants were seated comfortably at a distance of 1.15 m from a 19-inch LCD computer monitor in a soundproof booth. The raw EEG was recorded with 64 Ag-AgCl electrodes attached to an elastic cap at the sampling rate of 1000 Hz by the NeuroScan system (Compumetics NeuroScan^®^, Victoria, Australia). All electrodes were placed according to the International 10–20 electrode placement standard with a ground electrode located at the AFz electrode, and the recording reference placed between Cz and CPz. Four bipolar facial electrodes were placed above and below the left eye and outer canthi of both the eyes to monitor vertical and horizontal eye movements (EOG channels) and two electrodes were placed on two mastoids to be used offline for re-referencing. Electrode impedances were kept at or below 8 kΩ throughout the recording.

The EEG experiment was divided into two sessions (explicit or implicit). Each session contained two blocks (prosodic or semantic). In each block, 180 spoken words of different emotional prosody or semantics (60 happy, 60 sad, 60 neutral) were presented binaurally through E-A-R TONE™ 3A Insert Earphone at 70 dB SPL. For explicit emotion perception, participants were instructed to attend to the emotional information of the stimuli. They indicated whether a word was spoken with a happy, neutral or sad tone of voice (prosodic block), and whether a word conveyed happy, neutral or sad semantic content by pressing one of the three buttons (semantic block). For implicit emotion perception, participants were instructed to attend to the gender of the speaker while ignoring the emotional information of the words. They indicated whether the word was spoken by a male or female speaker by pressing one of the two buttons in both prosodic and semantic blocks. E-prime (version 2.0.10) was used for stimulus presentation [81]. The presentation order of the session, block and button press was counterbalanced across participants.

**Figure 1:**
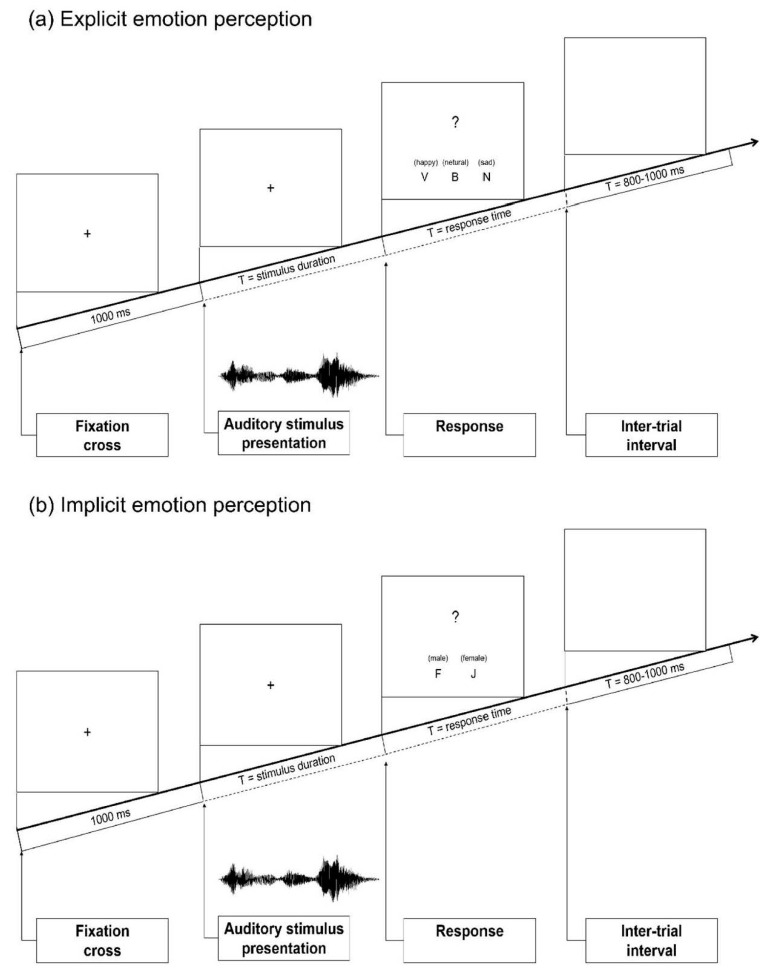
Schematic illustration of the experimental protocol for (**a**) explicit and (**b**) implicit emotion perception tasks.

Before each experimental block, participants were given a 10-trial training session and entered the experiment with at least 80% identification accuracy. There were 180 trials in each block. Each trial started with a fixation cross presented centrally on the screen for 1000 ms. The words were then presented auditorily, during which the fixation cross remained on the screen to minimize eye movements. Afterwards, a question mark was presented, which signaled the beginning of response. The words were presented in a pseudo-randomized manner. To reduce baseline artifacts, a variable inter-trial interval of 800–1000 ms occurred before the next trial began. A short pause of 10 s was provided after every 20 trials. There was a 2 min break between the two blocks in each session, and there was a 5 min break between the two sessions. The total duration of the experiment was approximately 60 min. During the experiment, behavioral (i.e., accuracy, reaction time) and electrophysiological data were recorded. The schematic illustration of the experimental protocol is presented in Figure 1.

### 2.4. Data Analysis

*ERP data analysis*. EEG data processing was performed with Matlab-based (Version: R2016a) EEGLAB (Version: 14.1.2) and ERPLAB (Version: 7.0) toolboxes. Only trials with correct behavioral responses were included in the ERP waveform and time-frequency (TF) analysis. The raw EEG data were down-sampled to 250 Hz. Eye blinks and muscle movements were identified and removed using Independent Component Analysis (ICA) algorithm following the guidelines by Chaumon et al. [82]. Artifact detection was performed according to the following criteria: (i) the maximally allowed amplitude difference for all EEG channels within a moving window (width: 200 ms; step: 50 ms) should not exceed ± 30 μV; (ii) the maximally allowed absolute amplitude for all EEG channels throughout the whole epoch should not exceed ± 100 μV. After excluding trials with incorrect responses and rejecting artifact-contaminated trials, the overall data retention rate was 95.1%. The data were re-referenced to the algebraic average of the two mastoid electrodes.

**Figure 2:**
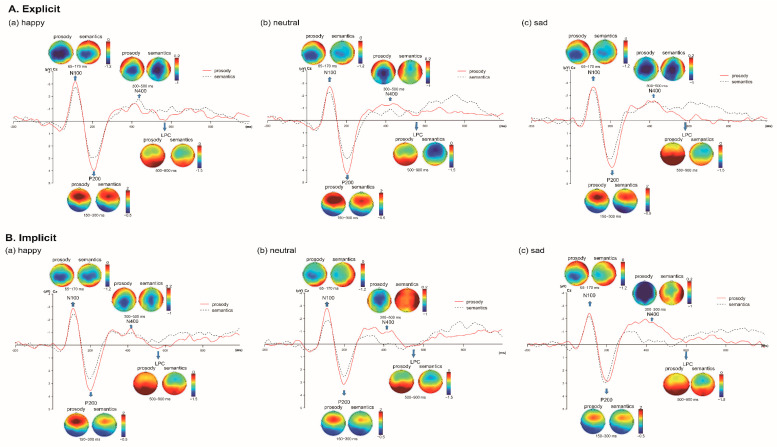
Grand averaged waveforms at Cz and topographical maps of mean amplitude in the N100, P200, N400 and LPC windows for (**a**) happy, (**b**) neutral and (**c**) sad stimuli in prosodic and semantic channels across (**A**) explicit and (**B**) implicit tasks.

For the auditory ERP analysis, the EEG data were band-passed at 0.1–40 Hz, and were segmented into time-based epochs of 1200 ms, which consisted of a 200 ms pre-stimulus interval for baseline correction and a 1000 ms post-stimulus interval. Grand average ERP waveforms (Figure 2) were computed for each emotion (happy, neutral and sad) in each channel (semantic vs. prosodic) under each task (explicit vs. implicit). Four time windows were chosen for analyses based on previous literature and visual inspection of the grand mean auditory ERP data (i.e., N100: 65–170 ms; P200: 150–300 ms; N400: 300–500 ms; LPC:500–900 ms) [38,39,40,42,43,73]. Since maximal effects were observed at the fronto-central and central sites, we selected six electrodes (FC3, FCz, FC4, C3, Cz, C4) for statistical analyses, which was consistent with previous reports [38,39,43,83]. The amplitude data were quantified by averaging data points within the time window of 40 ms around the peak of the components for each condition.

For the TF analysis, we evaluated two measures of cortical oscillations in delta (1–3.9 Hz), theta (4–7.9 Hz) and alpha (8–11.9 Hz) frequency bands at electrode Cz, namely, inter-trial phase coherence (ITPC) and event-related spectral perturbation (ERSP). ITPC estimates the trial-by-trial synchronization as a function of time and frequency, the value of which in a given frequency band can range from 0 to 1. Larger ITPC values indicate better trial-by-trial synchronization, and smaller values suggest lower consistency or larger neural “jittering” across trials. ERSP suggests trial-by-trial changes in spectral power (in dB) from pre-stimulus baseline as a function of time and frequency [22].

The two measures were computed using the “newtimef” function with the open-source EEGLAB package [84]. A modified short-term Fourier Transform (STFT) with Hanning window tapering was implemented to extract the ITPC and ERSP values for the delta, theta, and alpha frequency bands, which is recommended for the analysis of low-frequency activities. Zero-padding was applied to short epochs that did not have sufficient number of sample points with a padratio of 16 for Fourier transform. Frequencies for ITPC and ERSP calculation ranged from 0.5 to 50 Hz with a step interval of 0.5 Hz. An epoch window of 1800 ms with an 800 ms pre-stimulus baseline was used. The maximum ITPC and ERSP values in the designated time windows of N100 (65–170 ms), P200 (150–300 ms), N400 (300–500 ms) and LPC (500–900 ms) were identified per participant for each emotion category in each channel under each task for statistical analyses.

Statistical analyses of the event-related potential and TF data were conducted using linear mixed-effect (LME) models in R (version 4.0.3) [85]. For the waveform analysis, N100, P200, N400 and LPC amplitudes were analyzed as dependent variables, respectively. For the TF analysis, the delta, theta and alpha ITPC and ERSP in the corresponding time windows of the two components were entered as dependent variables, respectively. Within-subject factors included communication channel (semantic and prosodic), emotion category (happy, neutral and sad), and task type (explicit and implicit). The semantic channel, the sad emotion, and the implicit task were set as the baseline level for communication channel, emotion category, and task type, respectively. When happy stimuli were compared with the neutral ones, neutrality was set as the baseline. Subject was included as a random factor for intercepts. In case of significant main effects or interactions, Tukey’s post hoc tests were carried out with the emmeans package [86]. Additionally, to examine the relationship between the auditory ERP and TF measures, LME models with ITPC and ERSP values as predictor variables were fit for N100, P200, N400 and LPC amplitudes. Delta, theta and alpha ITPC and ERSP were as entered as fixed effects, respectively, and subject was entered as a random effect for intercept. Two-tailed significance level with α = 0.05 was used for all statistical analyses throughout the study. The full model with intercepts, coefficients, and error terms for the analysis of each neurophysiological index is shown in Supplemental Materials (Part III). To control the false discovery rate (FDR), we applied the Benjamini-Hochberg FDR methods to adjust the *p*-values for each model [87]. In the following *Results* section, we reported the significant main effects of the three factors and the highest-level multivariate interaction with a focus on the prosodic vs. semantic contrasts. Analyses on the emotion-factor contrast in the two channels for the two tasks, and the task-contrasts for the three emotional conditions in the two tasks in Supplemental Materials (Table S11).

*Behavioral data analysis.* A three-way multivariate analysis of variance (MANOVA) was conducted in R (version 4.0.3) [85] to investigate the statistical significance of communication channel (prosodic or semantic), emotion category (happy, neutral or sad) and task type (explicit or implicit) on identification accuracy and reaction time. The semantic channel, the sad emotion, and the implicit task were set as the baseline level for communication channel, emotion category, and task type, respectively. When happy stimuli were compared with the neutral ones, neutrality was set as the baseline. To test the MANOVA assumption, we first carried out a Pearson correlation test, which suggested that the two outcome variables (i.e., accuracy and reaction time) were correlated (*r* = −0.25, *p* < 0.001). Then, the two behavioral measures were entered as dependent variables in MANOVA with Pillai’s trace statistics reported. For any significant differences in the MANOVA results, we followed up the analysis with univariate analyses of variance (ANOVA). Similarly, FDR adjustments on *p* value were conducted for each ANOVA model and Tukey’s post hoc tests were conducted to examine pairwise comparisons in case of a significant main effect or interaction in the univariate analyses of each individual outcome measure.

## 3. Results

### 3.1. Auditory Event-Related Potential Measures

**Figure 3:**
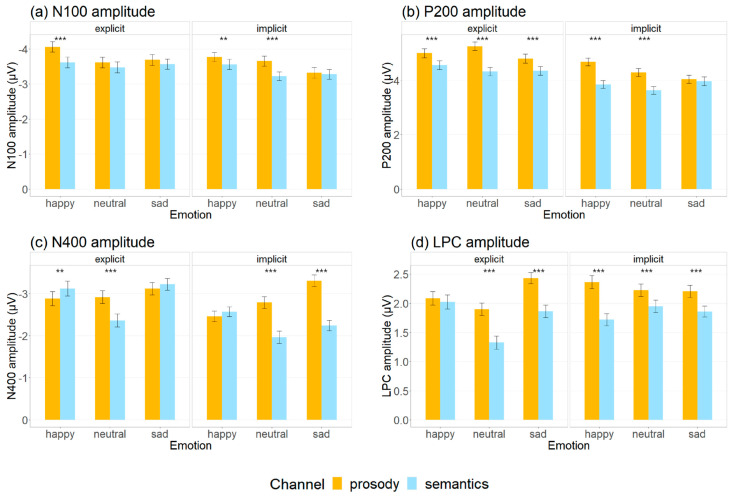
Bar plots of auditory ERP amplitude of (**a**) N100, (**b**) P200, (**c**) N400 and (**d**) LPC for happy, neutral and sad stimuli in prosodic and semantic channels across explicit and implicit tasks. Mean amplitude is displayed in the bar charts with error bars showing 95% confidence intervals. Asterisks mark the significance level: ** *p* < 0.01; *** *p* < 0.001.

The mean and standard deviation of N100, P200, N400 and LPC amplitudes (μV) elicited by happy, neutral and sad stimuli in prosodic and semantic channels across explicit and implicit tasks (Figure 2) are demonstrated in Table S7 and illustrated in Figure 3. Table 1 summarizes the effects that reached significance for the auditory ERP indices.

*N100.* LME analyses on N100 amplitudes revealed main effects of channel (χ^2^ (1) = 58.58, *p* < 0.001), emotion (χ^2^ (2) = 72.23, *p* < 0.001), and task (χ^2^ (1) = 43.63, *p* < 0.001). Post hoc multiple-comparison tests suggested that larger N100 amplitudes were observed for emotional prosody than emotional semantics ($\hat{\beta }$ = −0.23, *SE* = 0.03, *z* = −7.67, *p* < 0.001, *d* = −0.18), and for explicit tasks than the implicit ones ($\hat{\beta }$ = −0.20, *SE* = 0.03, *z* = −6.62, *p* < 0.001, *d* = −0.16). N100 was also increased for happy stimuli relative to the neutral ($\hat{\beta }$ = −0.26, *SE* = 0.04, z = −6.95, *p* < 0.001, *d* = −0.20) and sad ($\hat{\beta }$ = −0.29, *SE* = 0.04, *z* = −7.74, *p* < 0.001, *d* = −0.22) ones, while there was no significant difference between neutral and sad stimuli (*p* = 0.711). Significant interactions between channel and emotion (χ^2^ (2) = 12.65, *p* = 0.002) and between emotion and task (χ^2^ (2) = 9.33, *p* = 0.009) were found. More importantly, there was a significant three-way interaction among channel, emotion and task (χ^2^ (2) = 13.05, *p* = 0.003). Significantly increased N100 was found in emotional prosody compared with emotional semantics for happiness in both explicit ($\hat{\beta }$ = −0.44, *SE* = 0.07, *z* = −5.99, *p* < 0.001, *d* = −0.35) and implicit tasks ($\hat{\beta }$ = −0.21, *SE* = 0.07, *z* = −2.87, *p* = 0.004, *d* = −0.17) and for neutrality in implicit tasks ($\hat{\beta }$ = −0.43, *SE* = 0.07, *z* = −5.83, *p* < 0.001, *d* = −0.34). No significant prosody vs. semantic difference was found for sadness (explicit: *p* = 0.106; implicit: *p* = 0.550) and for neutrality in explicit tasks ($\hat{\beta }$ = −0.14, *SE* = 0.07, *z* = −1.91, *p* = 0.056, *d* = −0.11).

*P200.* LME analyses on P200 amplitudes showed main effects of channel (χ^2^ (1) = 267.71, *p* < 0.001), emotion (χ^2^ (2) = 29.81, *p* < 0.001), and task (χ^2^ (1) = 324.60, *p* < 0.001). Post hoc multiple-comparison tests suggested that larger P200 amplitudes were observed for emotional prosody than emotional semantics ($\hat{\beta }$ = 0.57, *SE* = 0.03, *z* = 16.51, *p* < 0.001, *d* = 0.39), and for explicit tasks than the implicit ones ($\hat{\beta }$ = 0.64, *SE* = 0.04, *z* = 18.22, *p* < 0.001, *d* = 0.43). P200 was also increased for happy stimuli relative to the neutral ($\hat{\beta }$ = 0.15, *SE* = 0.04, *z* = 3.43, *p* = 0.002, *d* = 0.10) and sad ($\hat{\beta }$ = 0.23, *SE* = 0.04, *z* = 5.4, *p* < 0.001, *d* = 0.16) ones, while there was no significant difference between neutral and sad stimuli (*p* = 0.119). More importantly, we observed a three-way interaction among channel, emotion and task (χ^2^ (2) = 24.45, *p* < 0.001). Significantly increased P200 was found in emotional prosody compared with emotional semantics for happy (explicit tasks: $\hat{\beta }$ = 0.44, *SE* = 0.08, *z* = 5.30, *p* < 0.001, *d* = 0.31; implicit tasks: $\hat{\beta }$ = 0.83, *SE* = 0.08, *z* = 9.96, *p* < 0.001, *d* = 0.58) and neutral (explicit tasks: $\hat{\beta }$ = 0.93, *SE* = 0.08, *z* = 11.17 *p* < 0.001, *d* = 0.65; implicit tasks: $\hat{\beta }$ = 0.66, *SE* = 0.08, *z* = 7.92, *p* < 0.001, *d* = 0.46) stimuli. For sad stimuli, P200 amplitudes were significantly larger in the prosodic channel (relative to the semantic one) in explicit tasks, and displayed a non-significant increasing trend in implicit tasks (explicit tasks: $\hat{\beta }$ = 0.45, *SE* = 0.08, *z* = 5.37, *p* < 0.001, *d* = 0.31; implicit tasks: *p* = 0.347).

*N400.* LME analyses on N400 amplitudes showed main effects of channel (χ^2^ (1) = 99.53, *p* < 0.001), emotion (χ^2^ (2) = 127.02, *p* < 0.001), and task (χ^2^ (1) = 127.04, *p* < 0.001). Post hoc analyses showed that larger N400 amplitudes were observed for emotional prosody than emotional semantics ($\hat{\beta }$ = −0.33, *SE* = 0.03, *z* = −10.01, *p* < 0.001, *d* = −0.24), and for the explicit task than the implicit one ($\hat{\beta }$ = −0.38, *SE* = 0.03, *z* = −11.32, *p* < 0.001, *d* = −0.27). N400 was also more negative for sad relative to happy ($\hat{\beta }$ = 0.21, *SE* = 0.04, *z* = 5.19, *p* < 0.001, *d* = 0.15) and neutral ($\hat{\beta }$ = 0.46, *SE* = 0.04, *z* = 11.31, *p* < 0.001, *d* = 0.33) stimuli, and more negative for happy relative to neutral stimuli ($\hat{\beta }$ = −0.25, *SE* = 0.04, *z* = −6.12, *p* < 0.001, *d* = −0.18). More importantly, the interaction among channel, emotion and task was also significant (χ^2^ (2) = 49.24, *p* < 0.001). Prosody elicited more negative N400 than semantics for neutral stimuli in explicit ($\hat{\beta }$ = −0.55, *SE* = 0.08, *z* = −6.94, *p* < 0.001, *d* = −0.40) and implicit ($\hat{\beta }$ = −0.82, *SE* = 0.08, *z* = −10.31, *p* < 0.001, *d* = −0.60) tasks, and for sad stimuli in the implicit task ($\hat{\beta }$ = −1.06, *SE* = 0.08, *z* = −13.28, *p* < 0.001, *d* = −0.77). Semantics triggered more negative N400 than prosody for happy stimuli in the explicit task ($\hat{\beta }$ = 0.24, *SE* = 0.08, *z* = 2.98, *p* = 0.003, *d* = −0.17). There was no significant difference between the two conditions for sad stimuli in the explicit task (*p* = 0.193) and for happy stimuli in the implicit task (*p* = 0.185).

*LPC*. LME analyses on LPC amplitudes showed main effects of channel (χ^2^ (1) = 242.33, *p* < 0.001), emotion (χ^2^ (2) = 61.53, *p* < 0.001), and task (χ^2^ (1) = 18.60, *p* < 0.001). Post hoc analyses showed that larger LPC amplitudes were observed for emotional prosody than emotional semantics ($\hat{\beta }$ = 0.41, *SE* = 0.03, *z* = 15.70, *p* < 0.001, *d* = 0.37), and for the implicit task than the explicit one ($\hat{\beta }$ = −0.12, *SE* = 0.03, *z* = −4.32, *p* < 0.001, *d* = −0.10). LPC was more positive for happy ($\hat{\beta }$ = 0.20, *SE* = 0.03, *z* = 6.09, *p* < 0.001, *d* = 0.18) and sad ($\hat{\beta }$ = −0.24, *SE* = 0.03, *z* = −7.35, *p* < 0.001, *d* = −0.21) relative to neutral stimuli, while no significant difference was found for happy and sad stimuli (*p* = 0.421). There was a significant interaction among channel, emotion and task (χ^2^ (2) = 58.30, *p* < 0.001). Prosody elicited more positive LPC amplitudes than semantics for happiness in implicit tasks ($\hat{\beta }$ = 0.64, *SE* = 0.06, z = 10.12, *p* < 0.001, *d* = 0.58), and for neutrality (explicit: $\hat{\beta }$ = 0.57, *SE* = 0.06, *z* = 8.96, *p* < 0.001, *d* = 0.52; implicit: $\hat{\beta }$ = 0.28, *SE* = 0.06, *z* = 4.38, *p* < 0.001, *d* = 0.25)) and sadness (explicit: $\hat{\beta }$ = 0.57, *SE* = 0.06, *z* = 8.99, *p* < 0.001, *d* = 0.52; implicit: $\hat{\beta }$ = 0.35, *SE* = 0.06, *z* = 5.44, *p* < 0.001, *d* = 0.31) in both types of tasks. No significant difference was found for happy stimuli in explicit tasks (*p* = 0.331).

### 3.2. Inter-Trial Phase Coherence Measures

**Figure 4:**
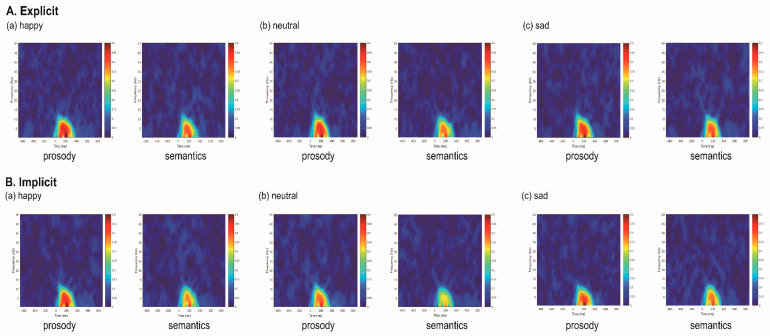
Time-frequency representations showing trial-to-trial phase-locking measured by ITPC for (**a**) happy, (**b**) neutral and (**c**) sad stimuli in prosodic and semantic channels across (**A**) explicit and (**B**) implicit tasks.

**Figure 5:**
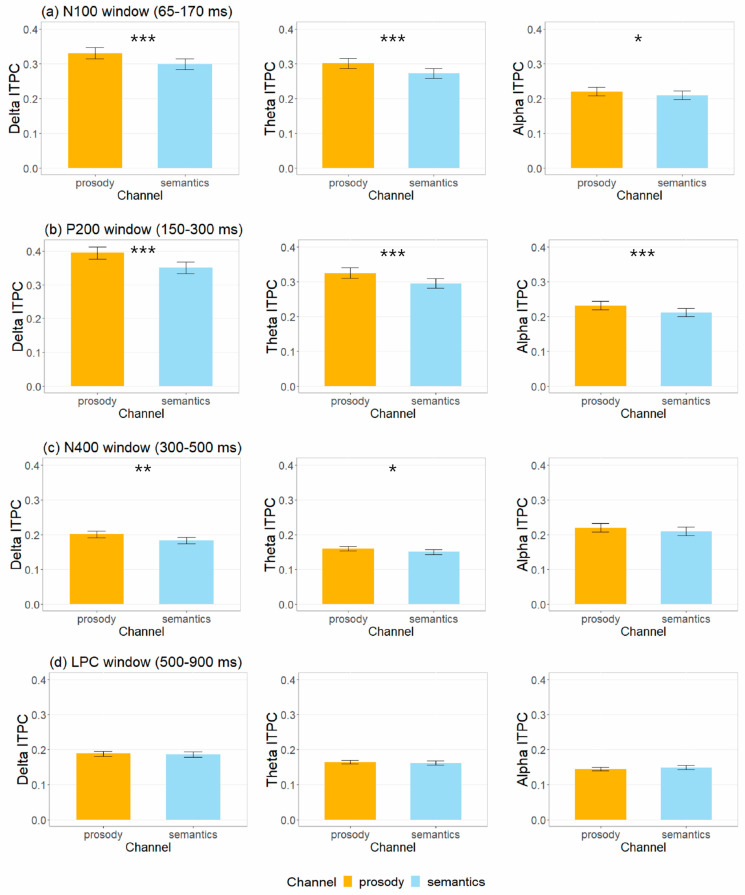
Bar plots of delta, theta and alpha ITPC associated with (**a**) N100, (**b**) P200, (**c**) N400 and (d) LPC for prosodic and semantic channels. Mean phase locking value is displayed in the bar charts with error bars showing 95% confidence intervals. Asterisks mark the significance level: * *p* < 0.05; ** *p* < 0.01; *** *p* < 0.001.

Figure 4 shows the time-frequency representations of trial-to-trial phase-locking measured by ITPC for happy, neutral and sad stimuli in prosodic and semantic channels across explicit and implicit tasks. The mean and standard deviation of delta, theta, and alpha ITPC values associated with N100, P200, N400 and LPC amplitudes are summarized in Table S8 and illustrated in Figure 5. Table 2 summarizes the effects that reached significance for the ITPC indices.

*N100.* In the N100 window, LME analyses on delta and theta ITPC showed main effects of channel (delta: χ^2^ (1) = 22.07, *p* < 0.001; theta: χ^2^ (1) = 24.67, *p* < 0.001), emotion (delta: χ^2^ (2) = 9.64, *p* = 0.019; theta: χ^2^ (2) = 10.65, *p* = 0.011) and task (delta: χ^2^ (1) = 20.05, *p* < 0.001; theta: χ^2^ (1) = 19.87, *p* < 0.001). Delta and theta ITPC values were larger in the explicit task than the implicit one (delta: $\hat{\beta }$ = 0.03, *SE* = 0.007, *z* = 4.54, *p* < 0.001, *d* = 0.48; theta: $\hat{\beta }$ = 0.03, *SE* = 0.006, *z* = 4.52, *p* < 0.001, *d* = 0.48), and in the prosodic channel than the semantic one (delta: $\hat{\beta }$ = 0.03, *SE* = 0.007, *z* = 4.75, *p* < 0.001, *d* = 0.50; theta: $\hat{\beta }$ = 0.02, *SE* = 0.006, *z* = 5.03, *p* < 0.001, *d* = 0.53). Happy stimuli produced greater delta and theta ITPC than the neutral (delta: $\hat{\beta }$ = 0.02, *SE* = 0.008, *z* = 2.85, *p* = 0.013, *d* = 0.37; theta: $\hat{\beta }$ = 0.02, *SE* = 0.007, *z* = 3.14, *p* = 0.005, *d* = 0.40) and sad (delta: $\hat{\beta }$ = 0.02, *SE* = 0.008, *z* = 2.51, *p* = 0.034, *d* = 0.32; theta: $\hat{\beta }$ = 0.02, *SE* = 0.007, *z* = 2.39, *p* = 0.046, *d* = 0.31) ones, while no significant difference was found for neutral and sad stimuli (*p* = 0.736). Analyses on alpha ITPC suggested a main effect of task (χ^2^ (1) = 10.00, *p* = 0.011). Alpha ITPC values were greater in the explicit than the implicit task ($\hat{\beta }$ = 0.02, *SE* = 0.005, *z* = 3.18, *p* = 0.002, *d* = 0.34).

*P200.* In the P200 window, LME analyses on delta ITPC exhibited main effects of channel (χ^2^ (1) = 45.06, *p* < 0.001), emotion (χ^2^ (2) = 7.86, *p* = 0.046) and task (χ^2^ (1) = 13.17, *p* < 0.001). There was increased delta ITPC in the prosodic than the semantic channel ($\hat{\beta }$ = 0.04, *SE* = 0.006, *z* = 6.91, *p* < 0.001, *d* = 0.73), and in the explicit than the implicit task ($\hat{\beta }$ = 0.03, *SE* = 0.007, *z* = 3.66, *p* < 0.001, *d* = 0.39). Happy stimuli produced greater delta ITPC values than the neutral one ($\hat{\beta }$ = 0.02, *SE* = 0.008, *z* = 2.69, *p* = 0.021, *d* = 0.35), while no significant difference was found between happy and sad stimuli and between neutral and sad ones (*p* > 0.05). Analyses on theta and alpha ITPC indicated main effects of channel (theta: χ^2^ (1) = 29.41, *p* < 0.001; alpha: χ^2^ (1) = 13.31, *p* = 0.002) and task (theta: χ^2^ (1) = 16.74, *p* < 0.001; alpha: χ^2^ (1) = 6.45, *p* = 0.039). Larger ITPC values were found in the prosodic than the semantic channel (theta: $\hat{\beta }$ = 0.02, *SE* = 0.006, *z* = 5.51, *p* < 0.001, *d* = 0.58; alpha: $\hat{\beta }$ = 0.02, *SE* = 0.005, *z* = 3.66, *p* < 0.001, *d* = 0.39) and in the explicit than the implicit task (theta: $\hat{\beta }$ = 0.02, *SE* = 0.006, *z* = 4.14, *p* < 0.001, *d* = 0.44; alpha: $\hat{\beta }$ = 0.01, *SE* = 0.005, *z* = 2.55, *p* = 0.011, *d* = 0.27).

*N400*. In the N400 window, LME analyses revealed main effects of channel for delta ITPC (χ^2^ (1) = 9.92, *p* = 0.011). Prosody elicited higher delta ITPC ($\hat{\beta }$ = 0.02, *SE* = 0.006, *z* = 3.16, *p* = 0.002, *d* = 0.33) than semantics.

*LPC*. In the LPC window, no significant main effect or interaction was found for delta, theta or alpha ITPC (all *p* > 0.05).

### 3.3. Event-Related Spectral Perturbation Measures

**Figure 6:**
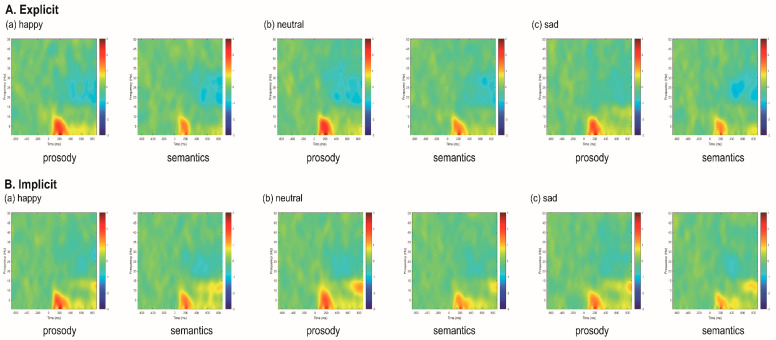
Event-related spectral perturbation (ERSP) for (**a**) happy, (**b**) neutral and (**c**) sad stimuli in prosodic and semantic channels across (**A**) explicit and (**B**) implicit tasks.

**Figure 7:**
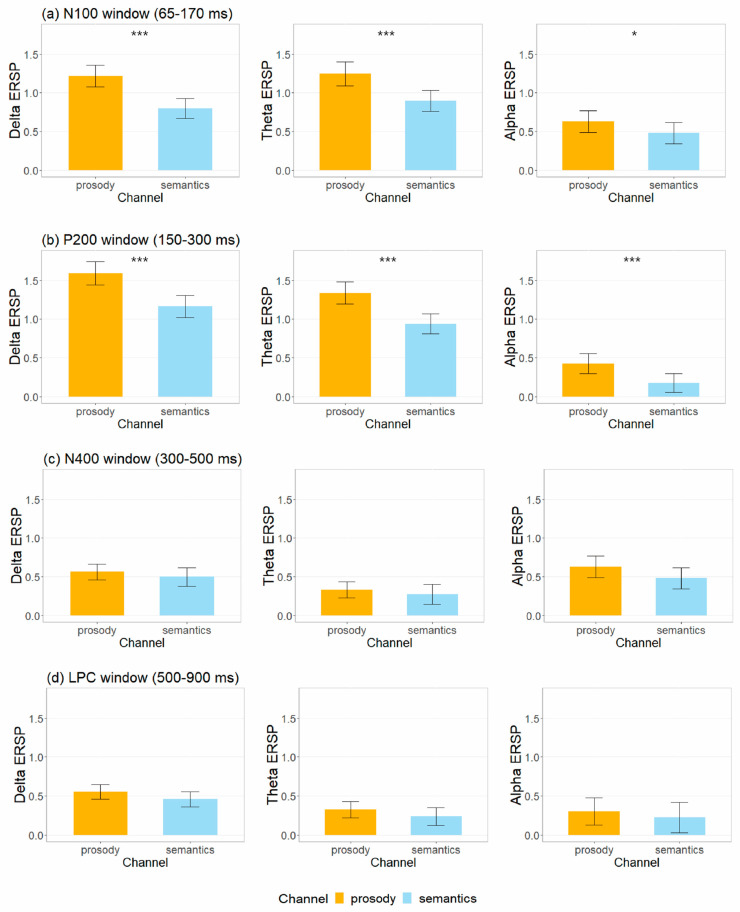
Bar plots of delta, theta and alpha ERSP associated with (**a**) N100, (**b**) P200, (**c**) N400 and (**d**) LPC for prosodic and semantic channels. Mean ERSP values are displayed in the bar charts with error bars showing 95% confidence intervals. Asterisks mark the significance level: * *p* < 0.05; *** *p* < 0.001.

Figure 6 shows the ERSP for happy, neutral and sad stimuli in prosodic and semantic channels across explicit and implicit tasks. The mean and standard deviation of delta, theta, and alph ERSP values associated with N100, P200, N400 and LPC amplitudes are summarized in Table S9 and illustrated in Figure 7. Table 3 summarizes the effects that reached significance for the ERSP indices.

*N100*. LME analyses revealed main effects of channel for delta (χ^2^ (1) = 39.61, *p* < 0.001) and theta (χ^2^ (1) = 28.04, *p* < 0.001) ERSP, and a main effect of task for theta (χ^2^ (1) = 9.20, *p* = 0.008) ERSP. Explicit tasks produced larger theta ($\hat{\beta }$ = 0.21, SE = 0.068, z = 3.05, *p* = 0.003, d = 0.32) ERSP than implicit tasks. Prosody triggered larger delta ($\hat{\beta }$ = 0.42, SE = 0.065, z = 6.45, *p* < 0.001, d = 0.68) and theta ($\hat{\beta }$ = 0.35, SE = 0.065, z = 5.38, p < 0.001, d = 0.57) ERSP than semantics. 

*P200*. LME models revealed main effects of task for theta (χ^2^ (1) = 4.98, p = 0.026) ERSP and main effects of channel for delta (χ^2^ (1) = 39.74, *p* < 0.001), theta (χ^2^ (1) = 36.66, *p* < 0.001) and alpha (χ^2^ (1) = 11.16, *p* = 0.006) ERSP. Explicit tasks produced larger theta ($\hat{\beta }$ = 0.15, SE = 0.069, z = 2.24, *p* = 0.026, d = 0.24) ERSP than implicit tasks. Prosody triggered larger delta ($\hat{\beta }$ = 0.43, SE = 0.066, z = 6.46, *p* < 0.001, d = 0.68), theta ($\hat{\beta }$ = 0.40, SE = 0.065, z = 6.19, *p* < 0.001, d = 0.65) and alpha ($\hat{\beta }$ = 0.25, SE = 0.07, z = 3.35, *p* < 0.001, d = 0.35) ERSP than semantics. 

*N400*. LME analyses revealed a main effect of task for delta (χ^2^ (1) = 10.53, *p* = 0.008) ERSP. Explicit tasks produced smaller delta ($\hat{\beta }$ = −0.23, SE = 0.069, z = −3.27, *p* = 0.001, d = −0.34) ERSP than implicit tasks.

*LPC*. LME analyses revealed main effects of task for alpha (χ^2^ (1) = 32.16, *p* < 0.001) ERSP. Explicit tasks produced smaller alpha ($\hat{\beta }$ = −0.48, SE = 0.082, z = −5.80, *p* < 0.001, d = −0.48) ERSP than implicit tasks.

### 3.4. Relationships between Auditory ERP and Neural Oscillation Indices

LME analyses revealed that delta (χ^2^ (1) = 48.01, *p* < 0.001) and alpha (χ^2^ (1) = 6.88, *p* = 0.013) ITPC were correlated with N100 amplitudes. In addition, delta (χ^2^ (1) = 133.15, *p* < 0.001) and theta (χ^2^ (1) = 17.17, *p* < 0.001) ITPC were significantly correlated with P200 amplitudes. N400 amplitudes were significantly correlated with delta ITPC (χ^2^ (1) = 7.94, *p* = 0.014). LPC amplitudes were correlated with delta (χ^2^ (1) = 13.49, *p* < 0.001) and theta ITPC (χ^2^ (1) = 6.58, *p* = 0.015). For these significant effects, higher ITPC values were significantly associated with stronger N100, P200, N400 and LPC enhancement (Table 4).

LME analyses also revealed that delta (χ^2^ (1) = 22.17, *p* < 0.001) ERSP was correlated with N100 amplitudes. Delta ERSP were correlated with P200 amplitudes (χ^2^ (1) = 62.60, *p* < 0.001), and alpha ERSP were correlated with LPC amplitudes (χ^2^ (1) = 6.86, *p* = 0.026). For these significant effects, greater ERSP were significantly associated with stronger N100, P200 and LPC enhancement (Table 4).

### 3.5. Behavioral Results

**Figure 8:**
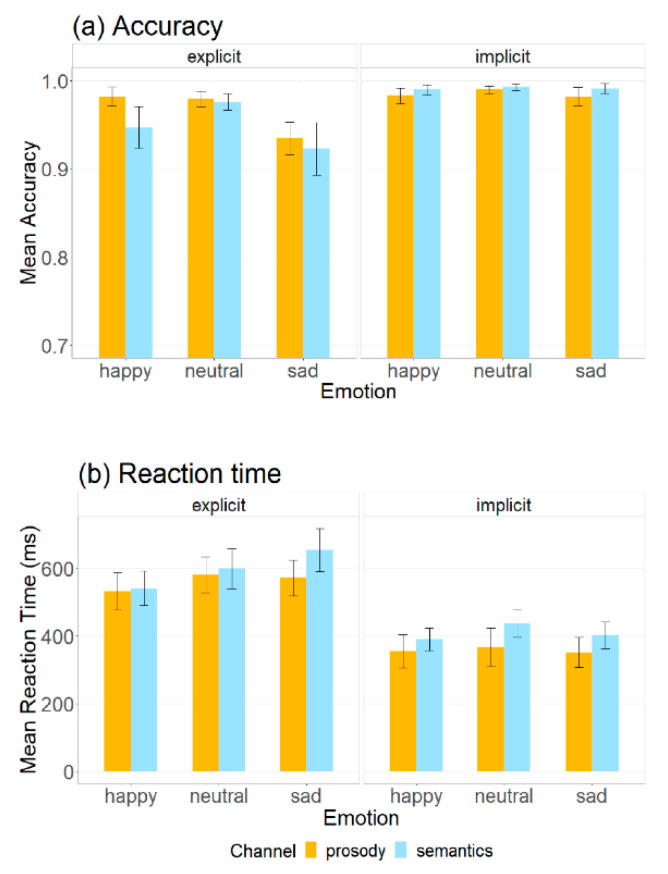
Identification (**a**) accuracy and (**b**) reaction time of happy, neutral and sad stimuli in prosodic and semantic channels across explicit and implicit tasks. Mean accuracy is displayed in the bar charts with error bars showing 95% confidence intervals.

Identification accuracy and reaction time data of happy, neutral and sad stimuli in prosodic and semantic channels across explicit and implicit tasks are summarized in Table S10 and visualized in Figure 8. Table 5 summarizes the effects that reached significance for the behavioral indices together with main findings of the neural data.

When analyzing the behavioral data, we excluded responses over two standard deviations from the mean reaction time (3.4%) [88]. Results of MANOVA indicated main effects of channel (Pillai’s trace = 0.03, *F* (2, 347) = 5.53, *p* = 0.007), emotion (Pillai’s trace = 0.10, *F* (4, 696) = 9.50, *p* < 0.001), and task (Pillai’s trace = 0.41, *F* (2, 347) = 122.04, *p* < 0.001), and significant interactions between emotion and task (Pillai’s trace = 0.07, *F* (4, 696) = 6.64, *p* < 0.001) and between channel and task (Pillai’s trace = 0.02, *F* (2, 347) = 4.44, *p* = 0.018) on accuracy and reaction time.

Separate univariate ANOVAs on accuracy data revealed a main effect of emotion (*F* (2, 348) = 15.79, *p* < 0.001). Post hoc multiple-comparison tests indicated that happy ($\hat{\beta }$ = 0.02, *standard error (SE)* = 0.005, z = 3.66, *p* < 0.001, *Cohen’s d* = 0.47) and neutral ($\hat{\beta }$ = 0.03, *SE* = 0.005, z = 5.51, *p* < 0.001, *d* = 0.71) stimuli triggered more accurate responses than the sad ones. There was no significant difference between happy and neutral stimuli (*p* = 0.157). In addition, there was a main effect of task (*F* (1, 348) = 61.32, *p* < 0.001). Explicit tasks produced less accurate responses than the implicit ones ($\hat{\beta }$ = −0.03, *SE* = 0.004, *z* = −7.81, *p* < 0.001, *d* = −0.82). More importantly, significant interactions between emotion and task (*F* (2, 348) = 11.75, *p* < 0.001) and between channel and task (*F* (1, 348) = 8.55, *p* = 0.006) were found. In explicit tasks, happy ($\hat{\beta }$ = 0.04, *SE* = 0.007, z = 5.20, *p* < 0.001, *d* = 0.95) and neutral ($\hat{\beta }$ = 0.05, *SE* = 0.007, *z* = 7.11, *p* < 0.001, *d* = 1.30) stimuli elicited more accurate responses than the sad ones, and there was no significant difference between happy and neutral stimuli (*p* = 0.138). In addition, emotional prosody yielded more accurate responses than semantics when attention was focused on the emotional aspect of the stimuli ($\hat{\beta }$ = 0.02, *SE* = 0.006, *z* = 3.00, *p* = 0.003, *d* = 0.45). In implicit tasks, however, there was no significant difference between any of the two emotional stimuli nor between the prosodic and semantic channels (all *p* > 0.05).

Separate univariate ANOVAs on reaction time revealed a main effect of channel (*F* (1, 348) = 9.54, *p* = 0.007). Emotional prosody elicited faster responses than semantics ($\hat{\beta }$ = −44.0, *SE* = 14.2, *z* = −3.09, *p* = 0.002, *d* = −0.33). There was no significant difference between neutral and sad stimuli (*p* = 0.998). Furthermore, a main effect of task was found (*F* (1, 348) = 188.88, *p* < 0.001). Explicit tasks produced slower responses than the implicit ones ($\hat{\beta }$ = 196, *SE* = 14.2, *z* = 13.73, *p* < 0.001, *d* = 1.45).

## 4. Discussion

The present study investigated how communication channelsspectralchannels, emo-tion categories and task types affected different stages of auditory emotional speech per-ception. We examined the auditory ERP responses, their corresponding oscillatory activi-ties and the behavioral performances elicited by spoken words expressing happiness, neutrality and sadness in either the prosodic or semantic channel under explicit and im-plicit emotion perception tasks. Overall, our neurophysiological and behavioral data re-vealed the modulatory role of channels, emotions, tasks and their reciprocal interactions in auditory emotion perception. Specifically, emotional prosody (relative to semantics) and happiness (relative to neutrality and sadness) are more perceptually dominant with greater neural activities during the sensory processing of acoustic signals and initial der-ivation of emotional significance, and better behavioral performance during cognitive evaluation of the stimuli. While explicit tasks also trigger greater neural responses than the implicit ones during early auditory processing, they produce reduced brain responses and poorer processing performance in the later stages. Interestingly, the prosodic domi-nance effect is meditated by emotional categories and task focuses, but the extent of mod-ulation is specific to different processing stages. In addition, our study indicated that os-cillation synchrony plays an important role in the neural generation of auditory event-related responses by showing increased ITPC and ERSP significantly correlated with enhanced auditory ERP amplitudes. These major findings will be discussed in detail in the following subsections.

### 4.1. Effects of Communication Channels on Emotional Speech Perception

Early auditory evoked potentials (i.e., N100 and P200) were identified for semantic and prosodic stimuli across participants, which indicates that both linguistic and para-linguistic emotion processing occurs before making judgments about the spoken stimuli [2,83,89,90]. These two types of information processing share some similarities in the time courses, which concurs with the three-stage model of emotion processing proposed by Schirmer and Kotz [37]. However, as predicted in Hypothesis 1, we observed important differences in the perceptual salience of the two communication channels: emotional prosody is consistently more perceptually salient than the semantic channel throughout emotional speech perception. It is generally assumed that early neurophysiological measures (e.g., N100, P200) primarily reflect sensory perception and late neurobehavioral measures (e.g., N400, LPC, accuracy, reaction time) demonstrate high-order cognitive pro-cessing. Our study shows that there was a general increase in all ERP amplitudes, neural oscillatory indices (esp. delta and theta ITPC and ERSP for N100, all ITPC and ERSP for P200, and delta ITPC for N400) as well as shorter reaction time for emotional prosody rel-ative to semantics. This suggests that prosody dominates over semantics not only during low-level sensory perception but also during high-level cognitive evaluation even when semantic processing is given more weight later on.

To our knowledge, this is the first study to provide neurophysiological evidence showing larger auditory evoked responses with smaller neural jittering and greater spec-tral power for the prosodic dominance effect during early and late emotional speech pro-cessing. The present study was also able to isolate the emotion processing in the re-sponse-making stage from the earlier perceptual and cognitive stages by measuring reac-tion time from the offset of auditory stimuli. The response time data demonstrated that prosody continues to dominate over semantics in the later decision-making stage, which replicates previous behavioral research on unisensory and multisensory emotion percep-tion in our lab [2,52,53,91]. The predominance of prosody over semantics can be related to differences in stimulus characteristics of the two channels. As shown in Tables S3, S4 and S6 in Supplemental Materials, prosodic stimuli showed greater variations in acoustic properties, including mean duration and f0, and emotional arousal among different emo-tional categories compared with the semantic ones, thus enjoying greater perceptual sali-ence throughout the three stages of emotion word processing. In addition, since our par-ticipants all spoke a tonal language (i.e., Mandarin-Chinese) as their mother tongue and lived in an East-Asian country with a high-context culture, they were likely to develop greater sensitivity to pitch-related cues that are important for prosody processing and rely heavily on contextual messages during social communication [15,92].Interestingly, the processing dominance of prosody over semantics are modulated by emotion categories and task types, though such modulatory effects are differentially represented at the three processing stages. The prosodic dominance effect was attenuated for sadness processing and in the implicit task during early auditory processing and decision-making. However, the effect was reduced for happiness processing in the explicit task during conscious emotion processing in the brain. Specifically, compared with emotional semantics, prosody elicited larger N100 amplitudes for happy and neutral stimuli but not for the sad ones in both explicit and implicit tasks. Larger P200 amplitudes were found in the prosodic channel for happy and neutral stimuli regardless of task focuses, but for sad stimuli in the explicit task only. However, this channel dominance effect was somewhat reduced and even displayed a reverse pattern (i.e., semantic dominance) during earlier stages of cognitive processing, as indexed by larger N400 amplitudes in the semantic channel when participants perceived happy stimuli in the explicit task. Larger LPC amplitudes were also observed for emotional prosody except for happiness processing in the explicit task, though there was a general increase in accuracy irrespective of emotion category when participants were guided to focus on the emotionality of prosody than that of semantics.

The differential representations of emotional and task modulation as time unfolds may be related to the distinct functions of each processing stage. In the context of early emotional speech processing, N100 reflects the physical features of the auditory stimuli, and P200 serves as an index of the emotional salience of a vocal stimulus [21,35,90]. In this perspective, sad stimuli in the present study were characterized by longer mean duration and lower mean f0 compared with the happy and neutral ones (Tables S3 and S4 in Supplemental Materials), which makes it difficult to differentiate the two communication channels for sadness processing irrespective of task requirements in the N100 window. In the P200 window, the prosodic dominance effect reached significance in explicit emotion identification tasks, while it only displayed a non-significant trend for the processing of sadness in implicit tasks. This implies that attention directed towards the emotional meaning of the stimuli plays a facilitatory role in the derivation of emotional significance from prosodic cues. Higher identification accuracy of prosodic stimuli in the explicit tasks but not in the implicit ones further suggests that task focuses not only shape early emotional speech perception but continue to interact with the channel dominance effect in the response-making stage of emotion processing. This finding is not surprising as in the implicit task, participants relied on similar vocal cues (esp. f0) for the perception of speaker’s gender in both channels [93]. By contrast, while they counted on various acoustic features (e.g., f0, duration, voice quality) to determine the emotional information of prosodic stimuli, they conducted higher-order semantic analyses to determine that of verbal content, which made the two channels more distinguishable in the explicit task. Moreover, late components such as N400 and LPC are more sensitive to lexico-semantic processing than earlier sensory components [94,95], which may explain why we observed reduced prosodic salience and even a reverse pattern of channel dominance favoring semantics especially when participants focused their attention on signals that contained incongruent information (e.g., happy words spoken in a neutral prosody).

### 4.2. Effects of Emotion Categories on Emotional Speech Perception

One important question centering around the effect of emotion is whether emotional signals can be differentiated from the neutral ones in speech processing [36,37,54]. Some differences were identified between the emotional and non-emotional signals in the present study, but the strength of the emotionality effect tends to be valence-dependent. Consistent with previous neurophysiological and behavioral observations [83,96,97,98], happy stimuli were consistently more perceptually salient than the neutral ones, as reflected by significantly larger N100, P200, N400 and LPC amplitudes, greater delta and theta ITPC values in the N100 window, and greater delta ITPC values in the P200 window. However, sadness did not differ from neutrality in the N100 and P200 windows, but elicited significantly larger N400 and LPC amplitudes later on. This is understandable as these late components reflect a more elaborate building-up of emotional meaning [35]. Such results underline the idea that the emotional salience of happiness emerges from early sensory stages, whereas sadness does not manifest its emotional significance until high-order cognitive processing of the spoken stimuli. During the response-making stage, in line with previous behavioral results [71], the identification accuracy of neutral stimuli was significantly higher than that of the sad stimuli, and even slightly (but not significantly) higher than that of the happy stimuli, though these differences only occurred in explicit tasks. It is likely that while both emotional stimuli contained semantics-prosody incongruency (e.g., happy/sad semantics spoken in a neutral prosody or semantically neutral words spoken in a happy/sad prosody), neutral stimuli were always congruent in prosody and semantics, thus producing more accurate identification when participants focused their attention on the emotional content of the stimuli.

Another important finding consistent with our prediction in Hypothesis 2 was that there were significant neurobehavioral differences between specific emotion types. Compared with sadness, happiness tended to be more perceptually salient as it triggered larger N100 and P200 amplitudes, greater delta and theta ITPC values in the N100 window, higher accuracy and shorter reaction time compared with the sad ones. Our electrophysiological data suggest that the differentiation between emotional categories can start as early as around 100 ms, which might be attributable to differential acoustic and arousal characteristics of the two emotions [37,45,60,99]. For example, happiness is often characterized by a faster speech rate (shorter duration), higher intensity and mean f0, and higher emotional arousal compared to sadness, thereby triggering larger auditory ERP responses during the initial sensory and emotional decoding of the stimulus. As delta oscillations depend on the activity of motivational systems and reflect salience detection, and theta oscillations are involved in emotional regulation [55,100], better phase alignment of cortical oscillations in happiness processing implicates that happiness tends to be more motivationally and emotionally significant than sadness, which might also contribute to its sensory dominance. In addition, happiness continued to produce better identification performances compared with sadness during behavioral evaluation of the auditory stimulus, which supports the claims of a positive outlook and prosocial benevolent strategies in social communication [61].

### 4.3. Effects of Task Types on Emotional Speech Perception

In the present study, participants intentionally directed their attention to the emotional aspect of the stimuli in explicit tasks, while they paid attention to the non-emotional property (speaker’s gender) of the stimuli in implicit tasks. Our electrophysiological, time-frequency, and behavioral data confirmed the third hypothesis that explicit tasks triggered larger neural responses during earlier stages of auditory emotion perception but produced reduced brain activities and poorer behavioral performance during later cognitive processing. Previous studies demonstrated distinctive effects of attention on N100, P200 and N400, with increased attention producing more negative N100 and N400 but less positive P200 amplitudes [64,65,66]. While we observed enhanced N100 and N400 as an indication of increased attentiveness in explicit tasks, there was also an increase in P200 amplitudes when attention was guided towards the emotional characteristics of the stimulus in our study. The P200 following the N100 is often referred to as part of the N1-P2 complex in auditory processing and shares many characteristics with the preceding component [101]. Another plausible account is that N100, P200 and N400 are sensitive to cognitive efforts as increased processing demands lead to enhanced auditory ERP amplitudes [39,69]. Given the differential roles of required attentiveness and cognitive efforts in shaping the auditory ERP components, we speculate that the two effects may exert an additive effect on the more negative-going N100 and N400 component in explicit tasks; by contrast, they may counteract in affecting the P200 amplitude with task demands exerting a more decisive influence.

The nature and difficulty of different task types can also explain the neural oscillatory patterns and late cognitive processing performances observed in the current study [70,71,97,102]. All ITPC indices for N100 and P200 showed a significant enhancement in explicit emotion recognition tasks relative to the implicit condition. According to Weiss and Mueller [70], higher inter-trial phase coherence is often found during increased task complexity, which requires a higher level of neuronal cooperation or synchronization. In this regard, our ITPC data suggest increased synchrony of neuronal oscillations across trials in the explicit task requiring top-down control of attention on the emotional aspect of the stimuli, which is more cognitively demanding than the gender discrimination task. However, we remain cautious when drawing conclusions concerning the oscillation results since these time-frequency representations contained power all the way down to 0.1 Hz, which may reflect transient brain responses [103]. In addition, these ITPC data were associated with the ERP differences and could reflect task-induced changes in the power of oscillations or concurrent evoked responses instead of actual changes in the phase of the ongoing activity [104]. We were aware of the caveat of inter-trial phase coherence and thus applied spectral power analyses, which can provide more direct evidence for the oscillatory activities. Our study showed increased theta ERSP in the N100 and P200 time windows for the explicit task and increased delta ERSP for the implicit task in the N400 time window. This finding suggested that the two experimental paradigms produced different temporal dynamics of the low frequency synchronization. However, we observed a different synchronization pattern from a previous study on emotional face processing, which showed enhanced theta and delta synchronization in the implicit task during unconscious processing whereas increased synchronization in the explicit task during conscious stages of information processing [97]. This may stem from the differences in stimulus modality and emotion category between studies. As expected, the differences between task types continue to influence the cognitive processing of the auditory stimuli. The implicit task elicited more positive LPC than the explicit one. Since LPC is often considered as a possible variant of P300, a decline in amplitudes may indicate greater task difficulty in explicit emotional identification [72]. We also observed greater alpha power in the LPC window. This may indicate greater efforts to inhibit emotional processing during the gender identification tasks, as alpha synchronization plays an important role in the inhibition of task-irrelevant information [55]. There could also be a potential effect of task difficulty on peak latency of the ERP [105]. Thus, task effects deserve to be carefully examined or controlled in future work. Similarly, we found significantly better identification performances in both accuracy and reaction time measures in the implicit relative to the explicit task. It is conceivable that while the gender discrimination task was a binary (i.e., female vs. male) alternative forced-choice (AFC) task, the emotion recognition task involved differentiation among the three emotional categories (i.e., happy, neutral and sad), which automatically required more cognitive resources in memory retrieval and introduced more judgmental confounds in the response-making stage.

### 4.4. Neurophysiological and Behavioral Measures of Emotional Speech Perception

One noteworthy finding is that ITPC and ERSP values were significant predictors of auditory ERP amplitudes across experimental conditions, which supports our final hypothesis. Specifically, increased delta and alpha ITPC were correlated with more negative N100, increased delta and theta were related to more positive P200, increased delta was associated with more negative N400, and increased delta and theta was predictive of more positive LPC. Similarly, for the spectral power data, increased delta ERSP were correlated with more negative N100 and more positive P200, and increased alpha ERSP and were associated enhanced LPC. These patterns are consistent with findings from healthy [22,28] and clinical [23,25,27,77] populations. Although previous studies have examined whether ITPC and ERSP are able to predict variations in the obligatory N1-P2 complex response to speech sounds [106], very few studies have investigated whether measures of event-related cortical oscillations are potential indicators of auditory ERP responses (especially late components) using emotional speech stimuli. Therefore, our findings add to the extant literature in showing that trial-by-trial neural synchrony and spectral power contribute to the neural generation of auditory ERPs in early and late emotional speech processing [24,31].

It is noteworthy that different types of neurological activities and their subsequent behavioral performances did not always exhibit the same profile in characterizing emotional speech processing. For instance, while interaction effects among channels, emotions and tasks were observed for all auditory ERP components, no significant interplay was found among the three factors in the ITPC and ERSP measures. Moreover, there remained some distinctions even among the results from different indices belonging to the same type of experimental measure (e.g., waveform amplitudes in different time windows, ITPC and ERSP data of different frequency bands, or accuracy and reaction time as behavioral data). These differences in findings may be related to differential sensitivities to various measurement indices and processing stages [102,107]. Future work can further investigate in what measures, contexts, and processing stages the observed effects of channels, emotions and tasks can be generalized and in what conditions they may or may not be replicated, which will offer more refined ways to interpret the underlying mechanisms of emotional speech processing [53].

### 4.5. Implications, Limitations and Future Studies

The present study elucidates how the channel dominance effect, emotionality effect and task effect converge in shaping emotional speech processing, which sheds new light on the theoretical debates and underlying neural substrates and behavioral mechanisms of emotion cognition. Our findings contributed tonal language data from a high-context culture to the three-stage model of emotion cognition by delineating the temporal dynamics, neural oscillation characteristics and behavioral performances of emotional prosody and semantics processing in explicit and implicit emotion perception tasks. Apart from the three contextual factors explored in the current study, individual differences have also been repeatedly reported to influence emotion processing [37]. Future work can specify how the individual variables, including personality [108], age [109] and gender [74], can modulate emotional speech processing at different stages. Since we involved participants from a tonal language background and a high-context Chinese culture, the current work can also inspire new efforts to unravel the cross-linguistic and cross-cultural differences in emotion processing [110]. Furthermore, the current experimental protocol can be applied to testing clinical populations who reportedly display dysfunctions in auditory processing and emotion perception, such as cochlear implant users [111], individuals with schizophrenia [39,44], autism [112] and Parkinson disease [113], which can promote insightful understanding of the behavioral symptomology and underlying neural basis of the diseases.

Limitations of the current study need to be acknowledged. First, emotional information was conveyed through either the prosodic or semantic channel in our experiment. Though it is possible to communicate affective messages through a single channel (e.g., talking on the telephone or listening to news broadcast) in real-life settings [114], it is more often the case that emotions are expressed concurrently through auditory (e.g., prosody and semantics) and visual (e.g., facial expressions) channels in which congruent and incongruent information can be transmitted. Therefore, it is worthwhile to delve into the neural correlates of multisensory integration of emotions and investigate how different channels interact with one another in online emotion processing [115]. Second, findings might also be limited as we focused on two of the basic emotions (i.e., happiness, sadness) and neutrality in our study. Though such selection of emotions allowed us to compare voluntary and involuntary prosodic and semantic processing using emotional and non-emotional stimuli, it has led to some asymmetries in task difficulty between the explicit (three AFC) and implicit (two AFC) tasks as discussed earlier in the third subsection of *Discussion*. Future studies are encouraged to employ an experimental design with comparable complexity between tasks and explore whether the current findings can be extended to other categories of basic (e.g., anger, disgust, surprise, fear) and complex (e.g., embarrassment, guilt) emotions and required focuses of attention (e.g., emotional arousal of the stimuli or decoders) [45]. Third, we observed significant differences in brain responses between neutral prosody and semantics, which may be related to some intrinsic differences between the prosodic and semantic stimulus sets, such as the word frequency, word types (i.e., noun vs. adjectives) and word number (i.e., 60 different words for the prosodic set vs. 180 words for the semantic set). Other acoustic (e.g., f0, duration) and prosodic features (e.g., tonal combination) of the disyllabic speech stimuli may also lead to the observed differences between channels. It also seems difficult to make sure whether comparable amounts of valence were presented in each channel type. As such, it is possible that the larger ERP effects in the prosodic channel were due to more valenced stimuli used in that channel. Future studies are recommended to isolate the emotional aspect alone by controlling the potential confounds such as removing all the speech elements and presenting sound contours that differ in the same way between conditions, or using the exact same words (with or without emotional connotations) for testing different conditions. Fourth, we observed N400 amplitude differences in some conditions (e.g., implicit neutral and sad), which may affect the subsequent measure of LPC amplitudes. This is likely due to the design of our experiment, in which we divided our EEG session into two tasks (i.e., explicit or implicit) and each task contained two blocks (i.e., prosodic or semantic). Although the order of task and block was counterbalanced across participants, whether different orders led to differential amounts of repetition effect warrants further investigation. Moreover, we can see from the topographic maps in Figure 2 that the LPC effect was partially driven by some frontal negative responses to semantic conditions, so whether these are indeed LPC effects requires closer examination. The ERP methodology is limited in spatial resolution that is important for localizing the brain regions involved in generating scalp-recorded potentials [116]. Therefore, future studies combining ERP and functional magnetic resonance imaging techniques are needed to specify the engagement of brain structures involved in the time course of emotional speech processing [117].

## 5. Conclusions

The current work studied the interplay of channel, emotion and task effects on emotional speech processing using electrophysiological and behavioral measures. The results showed that prosody (relative to semantics) and happy stimuli (relative to the neutral and sad ones) gain more perceptual salience during the sensory processing of acoustic signals, initial derivation of emotional significance, and cognitive evaluation of the stimuli. Although the explicit emotion identification task tends to trigger greater neural responses compared to the implicit gender discrimination task during early processing stages, there is evidence for greater difficulty in task completion in the later decision-making stage. The channel salience effect over semantics tends to be emotion- and task-specific at different processing stages. In addition, stimulus-evoked phase alignment of oscillatory activity at different frequency bands plays a crucial role in generating the auditory event-related responses. Taken together, communication channel, emotion category and task focus interact to shape the time course, neural oscillations and behavioral activities of emotional speech processing, which enriches theoretical understanding of auditory emotion processing and provides the basis for further investigation on individual differences in emotion cognition from cross-cultural and clinical perspectives.

## Figures and Tables

**Table 1:** Effects that reached significance for auditory ERP and behavioral results.

	Factor	Channel	Emotion	Task	Condition * Emotion	Condition * Channel	Emotion * Channel	Condition * Emotion * Channel
Indice								
N100		χ^2^ = 58.58 ***	χ^2^ = 72.23 ***	χ^2^ = 43.63 ***	χ^2^ = 9.33 *	n.s.	χ^2^ = 12.65 **	χ^2^ = 13.05 **
P200		χ^2^ = 267.71 ***	χ^2^ = 29.81 ***	χ^2^ = 324.60 ***	χ^2^ = 15.49 ***	n.s.	χ^2^ = 42.86 ***	χ^2^ = 24.45 ***
N400		χ^2^ = 99.53 ***	χ^2^ = 127.02 ***	χ^2^ = 127.04 ***	χ^2^ = 7.45 *	χ^2^ = 62.33 ***	χ^2^ = 124.44 ***	χ^2^ = 49.24 ***
LPC		χ^2^ = 242.33 ***	χ^2^ = 61.53 ***	χ^2^ = 18.60 ***	χ^2^ = 97.46 ***	n.s.	n.s.	χ^2^ = 58.30 ***
Accuracy		n.s.	*F* = 15.79 ***	*F* = 61.32 ***	*F* = 11.75 ***	*F* = 8.55 **	n.s.	n.s.
Reaction time		*F* = 9.54 **	n.s.	*F* = 188.88 ***	n.s.	n.s.	n.s.	n.s.

Note. “n.s.” stands for not significant. Asterisks mark the significance level: * *p* < 0.05; ** *p* < 0.01; *** *p* < 0.001.

**Table 2:** Effects that reached significance for ITPC results.

Time Window	Frequency Band	Channel	Emotion	Task	Interaction
N100	Delta ITPC	χ^2^ = 22.07 ***	χ^2^ = 9.64 *	χ^2^ = 20.05 ***	No significant two-way or three-way interaction effects were found.
	theta ITPC	χ^2^ = 24.67 ***	χ^2^ = 10.65 *	χ^2^ = 19.87 ***	
	alpha ITPC	n.s.	n.s.	χ^2^ = 10.00 *	
P200	delta ITPC	χ^2^ = 45.06 ***	χ^2^ = 7.86 *	χ^2^ = 13.17 ***	
	theta ITPC	χ^2^ = 29.41 ***	n.s.	χ^2^ = 16.74 ***	
	alpha ITPC	χ^2^ = 13.31 **	n.s.	χ^2^ = 6.45 *	
N400	delta ITPC	χ^2^ = 9.92 *	n.s.	n.s.	
	theta ITPC	n.s.	n.s.	n.s.	
	alpha ITPC	n.s.	n.s.	n.s.	
LPC	delta ITPC	n.s.	n.s.	n.s.	
	theta ITPC	n.s.	n.s.	n.s.	
	alpha ITPC	n.s.	n.s.	n.s.	

Note. “n.s.” stands for not significant. Asterisks mark the significance level: * *p* < 0.05; ** *p* < 0.01; *** *p* < 0.001.

**Table 3:** Effects that reached significance for ERSP results.

Time Window	Frequency Band	Channel	Emotion	Task	Interaction
N100	Delta ERSP	χ^2^ = 39.61 ***	n.s.	n.s.	No significant two-way or three-way interaction effects were found.
	theta ERSP	χ^2^ = 28.04 ***	n.s.	χ^2^ = 9.20 **	
	alpha ERSP	χ^2^ = 4.41 *	n.s.	n.s.	
P200	delta ERSP	χ^2^ = 39.74 ***	n.s.	n.s.	
	theta ERSP	χ^2^ = 36.66 ***	n.s.	n.s.	
	alpha ERSP	χ^2^ = 11.16 ***	n.s.	n.s.	
N400	delta ERSP	n.s.	n.s.	χ^2^ = 10.53 **	
	theta ERSP	n.s.	n.s.	n.s.	
	alpha ERSP	n.s.	n.s.	n.s.	
LPC	delta ERSP	n.s.	n.s.	n.s.	
	theta ERSP	n.s.	n.s.	n.s.	
	alpha ERSP	n.s.	n.s.	χ^2^ = 32.16 ***	

Note. “n.s.” stands for not significant. Asterisks mark the significance level: * *p* < 0.05; ** *p* < 0.01; *** *p* < 0.001.

**Table 4:** Summary of LME models indicating the relationships between auditory ERP amplitude and neural oscillatory measures.

ERP Measure	Frequency Band	Chi-Square	Parameter Estimate	Standard Error	*t* Value	*p* Value
N100	Delta ITPC	48.01	−4.10	1.00	−4.09	<0.001
	Theta ITPC	1.30	0.51	1.37	0.38	0.255
	Alpha ITPC	6.88	−2.58	0.97	−2.65	0.013
	Delta ERSP	22.17	−0.32	0.07	−4.77	<0.001
	Theta ERSP	4.09	−0.22	0.11	−2.02	0.065
	Alpha ERSP	0.003	−0.005	0.09	−0.054	0.958
P200	Delta ITPC	133.15	5.27	1.02	5.16	<0.001
	Theta ITPC	17.17	3.48	1.51	2.30	<0.001
	Alpha ITPC	3.69	2.17	1.13	1.92	0.055
	Delta ERSP	62.60	0.62	0.07	8.38	<0.001
	Theta ERSP	2.76	0.09	0.21	0.40	0.097
	Alpha ERSP	3.51	−0.22	0.12	−1.87	0.091
N400	Delta ITPC	7.94	−2.84	1.01	−2.82	0.014
	Theta ITPC	2.75	−2.67	1.73	−1.66	0.146
	Alpha ITPC	0.97	−1.60	1.62	−0.99	0.324
	Delta ERSP	2.61	0.10	0.17	0.611	0.318
	Theta ERSP	0.02	0.06	0.21	0.303	0.900
	Alpha ERSP	0.27	−0.05	0.10	−0.516	0.901
LPC	Delta ITPC	13.49	3.04	0.82	3.71	<0.001
	Theta ITPC	6.58	3.47	1.35	2.58	0.015
	Alpha ITPC	0.44	−0.84	1.28	−0.66	0.509
	Delta ERSP	1.48	0.49	0.15	3.35	0.225
	Theta ERSP	3.36	−0.63	0.20	−3.20	0.100
	Alpha ERSP	6.86	0.22	0.08	2.62	0.026

**Table 5:** Summary of the effects of channel, emotion and task and their interactions in each neurophysiological and behavioral measure.

Stages	Early Stages: Basic Auditory Processing		Late Stages: Higher-Order Cognitive Processing			
Indices	N100	P200	N400	LPC	Behavioral Identification	
					Accuracy	Reaction Time
Main effect of channel	Pro > Sem (amplitude, delta and theta ITPC & ERSP)	Pro > Sem (amplitude, all ITPC & ERSP)	Pro > Sem (amplitude, delta ITPC)	Pro > Sem (amplitude)	Pro ≈ Sem	Pro < Sem
Main effect of emotion	Hap > Neu ≈ Sad (amplitude, delta and theta ITPC)	Hap > Neu ≈ Sad (amplitude) Hap > Neu (delta ITPC)	Sad > Hap > Neu (amplitude)	Hap ≈ Sad > Neu (amplitude)	Neu ≈ Hap > Sad	No main effect
Main effect of task	Exp > Imp (amplitude, all ITPC and theta ERSP)	Exp > Imp (amplitude, all ITPC theta ERSP)	Exp > Imp (amplitude) Exp < Imp (delta ERSP)	Exp < Imp (amplitude, alpha ERSP)	Exp < Imp	Exp > Imp
Interaction among factors	Pro > Sem not for sadness (amplitude)	Pro > Sem not for sadness in implicit tasks (amplitude)	Pro > Sem for neutrality in both tasks and for sadness for implicit tasks (amplitude) Sem > Pro for happiness in explicit tasks (amplitude)	Pro > Sem not for happiness in explicit tasks (amplitude)	Pro > Sem not for implicit tasks Neu ≈ Hap > Sad in explicit task only	Pro ≈ Sem

Notes. Pro = prosody; Sem = semantics; Hap = happy; Neu = neutral; Exp = explicit; Imp = implicit; “≈” indicates no significant differences. Index functions: N100 (Sensory processing of acoustic signals), P200 (Initial derivation of emotional meaning), N400 (Conflict processing and semantic integration), LPC (Conscious construction of emotional meaning).

## Data Availability

The datasets generated during and analyzed in the current study are available from the corresponding author on reasonable request.

## Supplementary Materials

The following supporting information can be downloaded at: https://www.mdpi.com/article/10.3390/brainsci12121706/s1, Figure S1: Spectral images of the (A) prosodic and (B) semantic stimuli for the (a) happy, (b) neutral and (c) sad emotions; Table S1: Words for the prosodic stimulus set; Table S2: Words for the semantic stimulus set; Table S3: Duration (milliseconds) of the experimental stimuli; Table S4: Mean f0 (Hertz) of the experimental stimuli; Table S5: Familiarity rating for the spoken words used in prosodic and semantic tasks; Table S6: Identification accuracy of emotional category and rating of emotional arousal for the experimental stimuli; Table S7: Mean amplitude (μV) of N100, P200, N400 and LPC elicited by happy, neutral and sad stimuli in prosodic and semantic channels across explicit and implicit tasks; Table S8: Delta, theta, and alpha ITPC measures in the windows of N100, P200, N400 and LPC elicited by happy, neutral and sad stimuli in prosodic and semantic channels across explicit and implicit tasks; Table S9: Delta, theta, and alpha ERSP measures in the windows of N100, P200, N400 and LPC elicited by happy, neutral and sad stimuli in prosodic and semantic channels across explicit and implicit tasks; Table S10: Mean identification accuracy and reaction time of happy, neutral and sad stimuli in prosodic and semantic channels across explicit and implicit tasks. Table S11: Emotion and task contrasts for the three-way interactions of auditory event-related potential amplitudes.
