# Supplementary material for: Neurocognitive Dynamics of Prosodic Salience over Semantics during Explicit and Implicit Processing of Basic Emotions in Spoken Words
Source: Brain Sci. 2022 Dec 12;12(12):1706. doi: 10.3390/brainsci12121706 (PMC9776349; doi:10.3390/brainsci12121706)
Supplement: Supplementary file 1 [file brainsci-12-01706-s001.zip › brainsci-2022581-supplementary.pdf]

Supplemental materials

Part I Table

Table S1. Words for the prosodic stimulus set.

咖啡<br>(coffee)	香蕉<br>(banana)	飞机<br>(airplane)	冰箱<br>(refrigerator)
蜻蜓<br>(dragonfly)	番茄<br>(tomato)	菠萝<br>(pineapple)	窗帘<br>(curtain)
斑马<br>(zebra)	钢笔<br>(pen)	书本<br>(book)	铅笔<br>(pencil)
风扇<br>(fan)	鸡蛋<br>(egg)	衣架<br>(hanger)	蟋蟀<br>(cricket)
台灯<br>(lamp)	牙刷<br>(toothbrush)	围巾<br>(scarf)	皮箱<br>(leather suitcase)
河流<br>(river)	蝴蝶<br>(butterfly)	白糖<br>(sugar)	篮球<br>(basketball)
毛毯<br>(blanket)	苹果<br>(apple)	啤酒<br>(beer)	牛奶<br>(milk)
肥皂<br>(soap)	茶叶<br>(tea)	名片<br>(business card)	邮票<br>(stamp)
水杯<br>(cup)	饼干<br>(biscuit)	纸巾<br>(tissue)	手机<br>(cell phone)
火柴<br>(match)	草莓<br>(strawberry)	纸牌<br>(card)	果皮<br>(peel)
手套<br>(glove)	草帽<br>(straw hat)	口罩<br>(mask)	假发<br>(wig)
面包<br>(bread)	闹钟<br>(alarm)	汽车<br>(car)	豆浆<br>(soy milk)
树林<br>(woods)	贝壳<br>(shell)	饭盒<br>(lunch box)	橡皮<br>(eraser)
报纸<br>(newspaper)	字典<br>(dictionary)	木桶<br>(barrel)	大米<br>(rice)
大象<br>(elephant)	电话<br>(telephone)	照片<br>(photo)	木棍<br>(stick)

Table S2. Words for the semantic stimulus set.

Happiness	Neutrality	Sadness
开心<br>(glad)	中间<br>(middle)	悲观<br>(pessimistic)
欢心<br>(exulted)	居中<br>(central)	悲哀<br>(mournful)
欢愉<br>(joyful)	均一<br>(even)	哀伤<br>(plaintive)
欣然<br>(pleased)	通俗<br>(popular)	心酸<br>(poignant)
欢腾<br>(rapturous)	通常<br>(routine)	伤心<br>(sad)
欢喜<br>(delighted)	中游<br>(middle reach)	忧伤<br>(mirthless)
舒坦<br>(comfortable)	中层<br>(middle-level)	悲伤<br>(downhearted)
欣喜<br>(blissful)	均匀<br>(uniform)	灰心<br>(discouraged)
兴奋<br>(excited)	相同<br>(identical)	揪心<br>(anxious)
欢乐<br>(gay)	平缓<br>(mild)	消极<br>(negative)
舒适<br>(snug)	基本<br>(fundamental)	凄凉<br>(dismal)
欢快<br>(cheerful)	基础<br>(basic)	哀愁<br>(grieved)
舒畅<br>(eased)	中立<br>(impartial)	消沉<br>(low-spirited)
高兴<br>(happy)	通用<br>(universal)	苍凉<br>(desolate)
欢悦<br>(joyous)	中性<br>(neutral)	忧愁<br>(woebegone)
安乐<br>(cosy)	相对<br>(relative)	心疼<br>(worried)
欣慰<br>(thankful)	相似<br>(similar)	悲凉<br>(dreary)
酣畅<br>(heartily)	相互<br>(mutual)	凄楚<br>(forlorn)
欢闹<br>(tumultuous)	中部<br>(middle)	酸楚<br>(distressed)
欢畅<br>(elated)	平均<br>(average)	凄惨<br>(tragic)
康乐<br>(healthy and happy)	折中<br>(compromised)	伤感<br>(sentimental)
微笑<br>(smile)	常规<br>(conventional)	悲苦<br>(sorrowful)
狂欢<br>(boisterous)	直接<br>(direct)	凄苦<br>(miserable)
和谐<br>(harmonious)	平常<br>(ordinary)	失落<br>(downcast)
祥和<br>(serene)	平凡<br>(undistinguished)	失望<br>(lamentable)
怡然<br>(enjoyable)	平时<br>(normal)	凄怆<br>(bleak)

吉祥<br>(auspicious)	平衡<br>(balanced)	悲怆<br>(despairing)
怡人<br>(nice)	平直<br>(straight)	悲痛<br>(heartrending)
明朗<br>(clear)	平时<br>(usual)	哀痛<br>(anguished)
甜美<br>(mellifluous)	寻常<br>(commonplace)	悲愤<br>(grieved and indignant)
狂喜<br>(thrilled)	持平<br>(same)	忧郁<br>(glum)
愉快<br>(merry)	垂直<br>(vertical)	心痛<br>(heart-broken)
融洽<br>(cordial)	平整<br>(smooth)	低落<br>(dejected)
得意<br>(jaunty)	平坦<br>(flat)	压抑<br>(repressed)
愉悦<br>(pleasurable)	平稳<br>(steady)	伤痛<br>(grievous)
甜蜜<br>(sweet)	同等<br>(equal)	忧虑<br>(apprehensive)
如愿<br>(fulfilled)	平淡<br>(bland)	忧闷<br>(morose)
陶醉<br>(intoxicated)	常见<br>(common)	愁苦<br>(melancholy)
满足<br>(fulfilled)	平日<br>(everyday)	沉痛<br>(heavyhearted)
美好<br>(glorious)	国际<br>(international)	惆怅<br>(disconsolate)
爽朗<br>(bright)	国内<br>(domestic)	颓丧<br>(crestfallen)
满意<br>(contented)	同类<br>(homogeneous)	难过<br>(upset)
有趣<br>(interesting)	普通<br>(plain)	难受<br>(uncomfortable)
爽快<br>(straightforward)	水平<br>(horizontal)	愁闷<br>(gloomy)
喜庆<br>(festive)	等同<br>(equivalent)	绝望<br>(desperate)
喜悦<br>(jubilant)	可能<br>(possible)	苦闷<br>(doleful)
乐观<br>(optimistic)	等距<br>(equidistant)	沮丧<br>(dispirited)
顺心<br>(satisfactory)	等量<br>(equivalent)	惨痛<br>(agonizing)
称心<br>(gratified)	一般<br>(general)	苦涩<br>(bitter)
自如<br>(free)	适中<br>(moderate)	扫兴<br>(disappointed)
快活<br>(jolly)	间接<br>(indirect)	痛心<br>(inconsolable)
幸福<br>(beatific)	日常<br>(daily)	痛惜<br>(regrettable)
豁达<br>(open-minded)	正常<br>(regular)	气馁<br>(demoralized)

痛快<br>(outright)	自然<br>(natural)	懊悔<br>(remorseful)
惬意<br>(agreeable)	共同<br>(joint)	懊恼<br>(annoyed)
畅快<br>(carefree)	静止<br>(static)	痛苦<br>(painful)
快乐<br>(happy)	适度<br>(appropriate)	郁闷<br>(depressed)
自在<br>(freely)	固定<br>(fixed)	暗淡<br>(dim)
乐意<br>(willing)	适当<br>(proper)	抑郁<br>(depressive)
庆幸<br>(lucky)	自动<br>(automatic)	丧气<br>(frustrated)

Table S3. Duration (milliseconds) of the experimental stimuli.

Stimulus type	Happy		Neutral		Sad		Mean	SD
	Mean	SD	Mean	SD	Mean	SD		
Prosody	1140.75	134.95	992.37	88.10	1626.77	187.24	1253.29	306.19
Semantics	999.28	78.57	995.12	72.44	997.96	72.47	997.45	74.57
Mean/SD	1072.16	131.81	993.70	80.90	1321.89	345.49	1129.25	259.50

Note. "SD" stands for standard deviation.

Table S4. Mean f0 (Hertz) of the experimental stimuli.

Stimulus type	Happy		Neutral		Sad		Mean	SD
	Mean	SD	Mean	SD	Mean	SD		
Prosody	237.95	37.41	144.47	36.25	137.74	30.22	173.38	57.13
Semantics	152.31	30.10	152.99	28.97	156.87	28.09	154.06	29.14
Mean/SD	196.42	54.70	148.60	32.33	147.02	30.73	164.01	46.76

Table S5. Familiarity rating for the spoken words used in prosodic and semantic tasks.

Stimulus type	Verbal content	Mean	SD
Prosody	semantically neutral concrete nouns	6.80	0.20
Semantics	adjectives with happy semantics	6.75	0.25
	adjectives with neutral semantics	6.76	0.25
	adjectives with sad semantics	6.71	0.28

Note. Participants rated the familiarity of the words on a 7-point scale (1 = not familiar, 7 = very familiar).

Table S6. Identification accuracy of emotional category and rating of emotional arousal for the experimental stimuli.

Stimulus type	Emotion category	Identification accuracy of emotional category		Rating of emotional arousal	
		Mean	SD	Mean	SD
Prosody	Happy	98.83%	2.48%	6.04	0.30
	Neutral	98.75%	2.17%	3.74	0.15
	Sad	99.50%	1.50%	1.64	0.23
Semantics	Happy	97.40%	3.80%	5.41	0.63
	Neutral	98.30%	2.60%	3.83	0.23
	Sad	98.50%	3.10%	1.97	0.27

Note. Participants identified the emotional category of the stimuli, and rated the emotional arousal on a 7-point scale (1= low, 7 = high).

Table S7. Mean amplitude (μV) of N100, P200, N400 and LPC elicited by happy, neutral and sad stimuli in prosodic and semantic channels across explicit and implicit tasks.

Measure	Task / Channel / Emotion	Explicit				Implicit			
		Prosody (Mean/SD)		Semantics (Mean/SD)		Prosody (Mean/SD)		Semantics (Mean/SD)	
N100	Happy	−4.06	1.79	−3.62	1.93	−3.77	1.63	−3.56	1.80
	Neutral	−3.62	1.89	−3.47	1.91	−3.66	1.74	−3.22	1.57
	Sad	−3.69	1.92	−3.57	1.82	−3.32	1.90	−3.28	1.74
P200	Happy	5.00	2.06	4.56	2.02	4.68	1.79	3.85	1.77
	Neutral	5.25	1.99	4.32	1.88	4.29	1.91	3.63	1.76
	Sad	4.80	2.07	4.35	1.95	4.04	1.94	3.96	2.03
N400	Happy	−2.88	2.09	−3.12	2.20	−2.46	1.56	−2.57	1.46
	Neutral	−2.92	1.87	−2.36	1.88	−2.79	1.74	−1.96	1.84
	Sad	−3.11	1.82	−3.22	1.74	−3.30	1.77	−2.24	1.58
LPC	Happy	2.09	1.45	2.02	1.53	2.36	1.41	1.72	1.29
	Neutral	1.90	1.32	1.33	1.40	2.23	1.30	1.95	1.30
	Sad	2.43	1.18	1.86	1.31	2.20	1.33	1.86	1.16

Table S8. Delta, theta, and alpha ITPC measures in the windows of N100, P200, N400 and LPC elicited by happy, neutral and sad stimuli in prosodic and semantic channels across explicit and implicit tasks.

Auditory ERP measure	Frequency band	Task / Channel / Emotion	Explicit				Implicit			
			Prosody (Mean/SD)		Semantics (Mean/SD)		Prosody (Mean/SD)		Semantics (Mean/SD)	
N100 (Mean/SD)	Delta	Happy	0.37	0.11	0.32	0.12	0.34	0.11	0.28	0.09
		Neutral	0.33	0.11	0.31	0.11	0.30	0.11	0.28	0.09
		Sad	0.33	0.11	0.32	0.10	0.31	0.10	0.28	0.08
	Theta	Happy	0.33	0.09	0.29	0.10	0.31	0.10	0.27	0.09
		Neutral	0.31	0.10	0.28	0.11	0.28	0.10	0.25	0.09
		Sad	0.30	0.11	0.29	0.10	0.28	0.08	0.27	0.09
	Alpha	Happy	0.23	0.09	0.22	0.08	0.22	0.11	0.20	0.07
		Neutral	0.23	0.08	0.21	0.10	0.22	0.08	0.20	0.08
		Sad	0.23	0.08	0.21	0.08	0.19	0.09	0.21	0.09
P200 (Mean/SD)	Delta	Happy	0.41	0.15	0.38	0.13	0.40	0.11	0.34	0.11
		Neutral	0.40	0.12	0.35	0.12	0.37	0.13	0.31	0.11
		Sad	0.40	0.12	0.37	0.12	0.39	0.12	0.35	0.12
	Theta	Happy	0.34	0.10	0.33	0.10	0.33	0.10	0.29	0.09
		Neutral	0.33	0.10	0.30	0.10	0.31	0.11	0.27	0.09
		Sad	0.33	0.10	0.31	0.11	0.31	0.10	0.29	0.09
	Alpha	Happy	0.25	0.09	0.22	0.08	0.23	0.10	0.21	0.07
		Neutral	0.24	0.09	0.21	0.09	0.23	0.08	0.20	0.09
		Sad	0.24	0.08	0.21	0.08	0.20	0.08	0.21	0.08
N400 (Mean/SD)	Delta	Happy	0.20	0.08	0.19	0.06	0.19	0.07	0.19	0.06
		Neutral	0.20	0.06	0.17	0.07	0.20	0.07	0.19	0.06
		Sad	0.20	0.06	0.18	0.06	0.21	0.07	0.18	0.07
	Theta	Happy	0.16	0.05	0.16	0.05	0.16	0.06	0.14	0.04
		Neutral	0.16	0.04	0.14	0.05	0.16	0.06	0.16	0.04
		Sad	0.16	0.04	0.15	0.04	0.16	0.04	0.15	0.05
	Alpha	Happy	0.14	0.04	0.13	0.03	0.14	0.04	0.11	0.05
		Neutral	0.13	0.04	0.12	0.04	0.13	0.03	0.13	0.04
		Sad	0.12	0.04	0.14	0.04	0.11	0.04	0.12	0.03
LPC	Delta	Happy	0.19	0.06	0.19	0.05	0.18	0.05	0.17	0.05

(Mean/SD)		Neutral	0.19	0.05	0.18	0.06	0.21	0.06	0.19	0.05
		Sad	0.17	0.04	0.19	0.05	0.18	0.04	0.19	0.04
		Happy	0.17	0.05	0.17	0.05	0.16	0.04	0.15	0.04
	Theta	Neutral	0.17	0.03	0.15	0.05	0.17	0.05	0.17	0.04
		Sad	0.16	0.03	0.17	0.04	0.15	0.04	0.16	0.03
		Happy	0.15	0.04	0.16	0.05	0.14	0.03	0.14	0.03
	Alpha	Neutral	0.14	0.04	0.14	0.03	0.14	0.03	0.15	0.04
		Sad	0.14	0.03	0.15	0.04	0.15	0.03	0.14	0.04

Table S9. Delta, theta, and alpha ERSP measures in the windows of N100, P200, N400 and LPC elicited by happy, neutral and sad stimuli in prosodic and semantic channels across explicit and implicit tasks.

Auditory ERP measure	Frequency band	Task / Channel / Emotion	Explicit				Implicit			
			Prosody (Mean/SD)		Semantics (Mean/SD)		Prosody (Mean/SD)		Semantics (Mean/SD)	
N100 (Mean/SD)	Delta	Happy	1.31	0.97	0.93	0.91	1.18	1.09	0.85	0.84
		Neutral	1.38	0.97	0.89	0.93	1.21	0.83	0.79	0.87
		Sad	1.26	1.00	0.79	0.80	0.96	0.86	0.54	0.88
	Theta	Happy	1.37	1.09	1.05	0.99	1.18	1.14	0.94	0.84
		Neutral	1.45	0.99	1.02	0.88	1.23	0.98	0.80	0.92
		Sad	1.28	1.09	0.89	0.94	0.98	1.03	0.68	0.90
	Alpha	Happy	0.70	0.91	0.63	1.03	0.52	0.94	0.58	0.83
		Neutral	0.68	0.94	0.54	1.07	0.85	1.00	0.43	0.87
		Sad	0.51	0.98	0.44	0.82	0.52	1.04	0.26	0.90
P200 (Mean/SD)	Delta	Happy	1.77	1.06	1.08	0.97	1.57	1.15	1.29	0.90
		Neutral	1.83	1.11	1.31	1.12	1.50	0.94	1.17	0.95
		Sad	1.55	1.13	1.10	1.01	1.34	0.73	1.04	0.88
	Theta	Happy	1.49	0.93	0.94	0.82	1.19	0.96	1.01	0.93
		Neutral	1.63	1.24	1.00	0.98	1.36	0.90	0.93	0.85
		Sad	1.27	1.02	0.96	0.87	1.09	0.75	0.78	0.74
	Alpha	Happy	0.44	0.65	0.22	0.78	0.24	0.88	0.26	0.75
		Neutral	0.44	1.08	0.07	1.00	0.71	0.86	0.31	0.81
		Sad	0.36	0.82	0.13	0.79	0.35	0.89	0.07	0.79
N400 (Mean/SD)	Delta	Happy	0.56	0.61	0.23	0.86	0.69	0.72	0.57	0.94
		Neutral	0.35	0.70	0.45	0.79	0.61	0.56	0.71	0.70
		Sad	0.61	0.68	0.30	0.73	0.55	0.86	0.73	0.76
	Theta	Happy	0.21	0.69	0.05	1.04	0.29	0.69	0.40	0.98
		Neutral	0.31	0.77	0.21	0.85	0.46	0.59	0.41	0.70
		Sad	0.39	0.75	0.12	0.76	0.32	0.76	0.44	0.78
	Alpha	Happy	−0.01	0.85	−0.21	1.23	0.07	1.08	0.45	1.24
		Neutral	0.25	1.05	0.06	1.06	0.22	0.86	0.02	0.94
		Sad	0.23	1.06	−0.04	1.20	0.29	1.06	0.01	1.07
LPC (Mean/SD)	Delta	Happy	0.59	0.71	0.41	0.62	0.54	0.69	0.51	0.67
		Neutral	0.44	0.67	0.41	0.81	0.65	0.53	0.48	0.53
		Sad	0.52	0.59	0.43	0.66	0.56	0.68	0.50	0.73
	Theta	Happy	0.28	0.65	0.07	0.73	0.29	0.79	0.40	0.77
		Neutral	0.26	0.80	0.12	0.94	0.46	0.51	0.30	0.70
		Sad	0.30	0.72	0.18	0.74	0.36	0.72	0.33	0.77
	Alpha	Happy	−0.04	0.98	−0.19	1.22	0.28	1.34	0.64	1.55
		Neutral	0.09	1.20	0.01	1.38	0.64	0.99	0.38	1.23
		Sad	0.30	1.11	−0.02	1.28	0.54	1.46	0.52	1.21

Table S10. Mean identification accuracy and reaction time of happy, neutral and sad stimuli in prosodic and semantic channels across explicit and implicit tasks.

Measure	Task / Channel / Emotion	Explicit				Implicit			
		Prosody (Mean/SD)		Semantics (Mean/SD)		Prosody (Mean/SD)		Semantics (Mean/SD)	
ACC	Happy	98.21%	2.87%	94.70%	6.29%	98.30%	2.34%	98.96%	1.47%
	Neutral	97.93%	2.34%	97.59%	2.48%	98.96%	1.19%	99.25%	0.98%
	Sad	93.48%	4.99%	92.30%	7.99%	98.17%	2.77%	99.11%	1.49%
RT	Happy	532.90	147.14	541.48	134.41	355.92	131.45	390.82	89.99
	Neutral	580.87	143.07	599.90	157.93	368.54	151.08	437.44	107.08
	Sad	572.34	140.49	654.54	169.75	352.81	120.02	402.99	104.76

Note. “ACC” stands for accuracy, and “RT” stands for reaction time.

Part II: Linear mixed-effects model

The full model with intercepts, coefficients, and error terms for the analysis of each neurophysiological measure is shown as follows:

$$\text{Each neurophysiological measure}_i = \beta_0 + (\beta_1 \times channel) + (\beta_2 \times emotion) + (\beta_3 \times task) + (\beta_4 \times channel \times emotion) + (\beta_5 \times channel \times task) + (\beta_6 \times emotion \times task) + (\beta_7 \times channel \times emotion \times task) + b_{0i} + \varepsilon_i$$

In the model, β_0 was the intercept, which represented the predicted value when all other factors were equal to 0. β_1, $\beta_2 \ldots \beta_7$ stood for the coefficients for communication channel, emotion category, task type and their interactions respectively. These coefficients suggested the extent to which the outcome variable changed relative to a unit of change in the corresponding predictors. The random intercepts were represented as b_{0i}, where i changed according to individual subjects. An error term (ε) was also added in the two models to indicate the distance between the predicted outcome and the actual data point (i.e., residual).

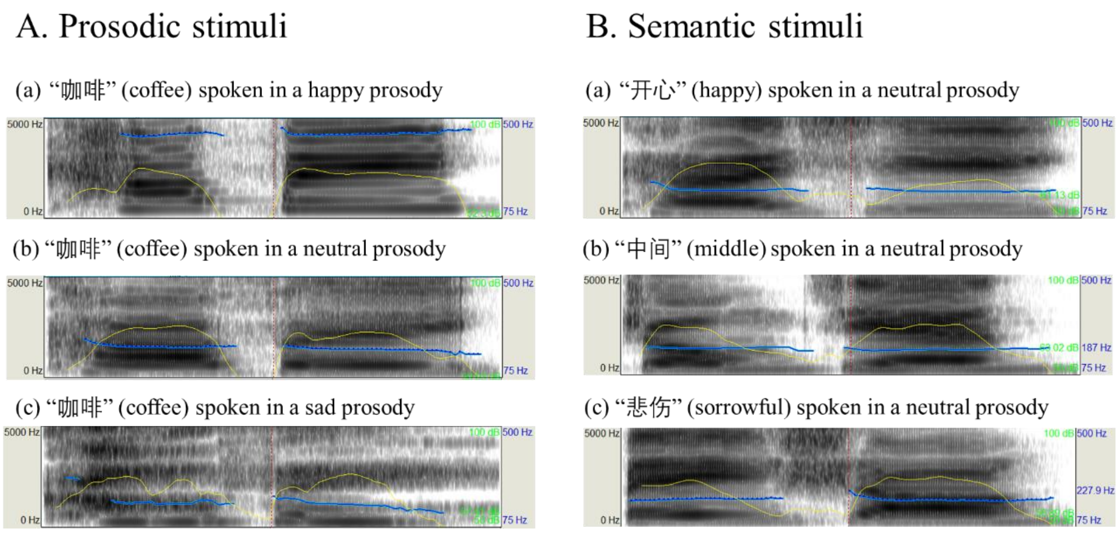

Figure S1. Spectral images of the (**A**) prosodic and (**B**) semantic stimuli for the (a) happy, (b) neutral and (c) sad emotions

Part III. Emotion and task contrasts for the three-way interactions of auditory event-related potential amplitudes

Table S11.

I.N100	contrast	estimate	SE	z.ratio	p.value
**1.Emotion contrast**					
channel=prosody,condition=explicit:	happy-neutral	−0.4447	0.0743	−5.986	<0.0001
	happy-sad	−0.3743	0.0743	−5.039	<0.0001
	neutral-sad	0.0704	0.0743	0.947	0.6104
channel=semantics,condition=explicit:	happy-neutral	−0.1421	0.0743	−1.912	0.1351
	happy-sad	−0.0496	0.0743	−0.668	0.7819
	neutral-sad	0.0924	0.0743	1.244	0.4271
channel=prosody,condition=implicit:	happy-neutral	−0.1162	0.0743	−1.564	0.2614
	happy-sad	−0.4508	0.0743	−6.069	<0.0001
	neutral-sad	−0.3346	0.0743	−4.505	<0.0001
channel=semantics,condition=implicit:	happy-neutral	−0.3361	0.0743	−4.524	<0.0001
	happy-sad	0.2819	0.0743	−3.795	0.0004
	neutral-sad	0.0542	0.0743	0.73	0.7459
**2.Task contrast**					
emotion=happy,channel=prosody:	explicit-implicit	−0.2895	0.0743	−3.897	0.0001
emotion=neutral,channel=prosody:	explicit-implicit	0.0391	0.0743	0.526	0.599
emotion=sad,channel=prosody:	explicit-implicit	−0.3659	0.0743	−4.926	<0.0001
emotion=happy,channel=semantics:	explicit-implicit	−0.058	0.0743	−0.781	0.4346
emotion=neutral,channel=semantics:	explicit-implicit	−0.2521	0.0743	−3.393	0.0007
emotion=sad,channel=semantics:	explicit-implicit	−0.2903	0.0743	−3.908	0.0001
**II.P200**					
**1.Emotion contrast**					
channel=prosody,condition=explicit:	happy-neutral	−0.2535	0.0836	−3.033	0.0068
	happy-sad	0.201	0.0836	2.405	0.0428
	neutral-sad	0.4545	0.0836	5.438	<0.0001
channel=semantics,condition=explicit:	happy-neutral	0.2375	0.0836	2.841	0.0125
	happy-sad	0.2068	0.0836	2.474	0.0356
	neutral-sad	−0.0307	0.0836	−0.367	0.9283
channel=prosody,condition=implicit:	happy-neutral	0.3868	0.0836	4.628	<0.0001
	happy-sad	0.6355	0.0836	7.603	<0.0001
	neutral-sad	0.2487	0.0836	2.976	0.0082
channel=semantics,condition=implicit:	happy-neutral	0.2162	0.0836	2.586	0.0263
	happy-sad	−0.1187	0.0836	−1.421	0.3302
	neutral-sad	−0.3349	0.0836	−4.007	0.0002
**2.Task contrast**					
emotion=happy,channel=prosody:	explicit-implicit	0.322	0.0836	3.849	0.0001
emotion=neutral,channel=prosody:	explicit-implicit	0.962	0.0836	11.51	<0.0001
emotion=sad,channel=prosody:	explicit-implicit	0.756	0.0836	9.048	<0.0001
emotion=happy,channel=semantics:	explicit-implicit	0.712	0.0836	8.516	<0.0001
emotion=neutral,channel=semantics:	explicit-implicit	0.69	0.0836	8.261	<0.0001
emotion=sad,channel=semantics:	explicit-implicit	0.386	0.0836	4.621	<0.0001
**III.N400**					
**1.Emotion contrast**					
channel=prosody,condition=explicit:	happy-neutral	0.0355	0.0799	0.445	0.8967
	happy-sad	0.2329	0.0799	2.915	0.01
	neutral-sad	0.1973	0.0799	2.47	0.036
channel=semantics,condition=explicit:	happy-neutral	−0.7566	0.0799	−9.471	<0.0001
	happy-sad	0.0991	0.0799	1.24	0.4295
	neutral-sad	0.8557	0.0799	10.712	<0.0001
channel=prosody,condition=implicit:	happy-neutral	0.3247	0.0799	4.065	0.0001
	happy-sad	0.8415	0.0799	10.534	<0.0001
	neutral-sad	0.5168	0.0799	6.469	<0.0001
channel=semantics,condition=implicit:	happy-neutral	−0.6046	0.0799	−7.568	<0.0001
	happy-sad	−0.3255	0.0799	−4.074	0.0001

	neutral-sad	0.2791	0.0799	3.494	0.0014
**2.Task contrast**					
emotion=happy,channel=prosody:	explicit-implicit	−0.42	0.0799	−5.252	<0.0001
emotion=neutral,channel=prosody:	explicit-implicit	−0.13	0.0799	−1.632	0.1027
emotion=sad,channel=prosody:	explicit-implicit	0.189	0.0799	2.367	0.0179
emotion=happy,channel=semantics:	explicit-implicit	−0.551	0.0799	−6.902	<0.0001
emotion=neutral,channel=semantics:	explicit-implicit	−0.399	0.0799	−4.999	<0.0001
emotion=sad,channel=semantics:	explicit-implicit	−0.976	0.0799	−12.216	<0.0001
**IV.LPC**					
**1.Emotion contrast**					
channel=prosody,condition=explicit:	happy-neutral	0.1892	0.0636	2.976	0.0082
	happy-sad	−0.3475	0.0636	−5.466	<0.0001
	neutral-sad	−0.5367	0.0636	−8.442	<0.0001
channel=semantics,condition=explicit:	happy-neutral	0.697	0.0636	10.965	<0.0001
	happy-sad	0.1622	0.0636	2.551	0.0289
	neutral-sad	−0.5349	0.0636	−8.413	<0.0001
channel=prosody,condition=implicit:	happy-neutral	0.1374	0.0636	2.161	0.0781
	happy-sad	0.1594	0.0636	2.507	0.0326
	neutral-sad	0.022	0.0636	0.346	0.9362
channel=semantics,condition=implicit:	happy-neutral	−0.2276	0.0636	−3.579	0.001
	happy-sad	−0.138	0.0636	−2.17	0.0764
	neutral-sad	0.0896	0.0636	1.409	0.336
**2.Task contrast**					
emotion=happy,channel=prosody:	explicit-implicit	−0.27794	0.0636	−4.372	<0.0001
emotion=neutral,channel=prosody:	explicit-implicit	−0.32979	0.0636	−5.188	<0.0001
emotion=sad,channel=prosody:	explicit-implicit	0.2289	0.0636	3.601	0.0003
emotion=happy,channel=semantics:	explicit-implicit	0.30334	0.0636	4.772	<0.0001
emotion=neutral,channel=semantics:	explicit-implicit	−0.62126	0.0636	−9.773	<0.0001
emotion=sad,channel=semantics:	explicit-implicit	0.00319	0.0636	0.05	0.9599
